# An interpretable machine learning model combining MRI-DKI habitat radiomic features and clinical biomarkers for noninvasive prediction of lymphatic metastasis in rectal cancer: a prospective study

**DOI:** 10.1186/s13244-026-02243-2

**Published:** 2026-03-25

**Authors:** Leping Peng, Feixiang Li, Fan Zhang, Fang Ma, Xiuling Zhang, Xiaoyue Zhang, Dongdong Chen, Gang Huang, Lili Wang

**Affiliations:** 1 Gansu University of Chinese Medicine, Lanzhou, China; 2 Department of Clinical and Technical Support, Philips Healthcare, Xi’an, China; 3 The Gastrointestinal Ward of General Surgery Department, Gansu Provincial Hospital, Lanzhou, China; 4 Department of Radiology, Gansu Provincial Hospital, Lanzhou, China

**Keywords:** Rectal cancer, Diffusion kurtosis imaging, Radiomics, Habitat, Lymphatic metastasis

## Abstract

**Objective:**

Tumor heterogeneity exerts a significant influence on lymphovascular invasion (LVI) and lymph node metastasis (LNM) in rectal cancer (RC), thereby affecting patient treatment outcomes and prognosis. This study aims to develop a combined model integrating diffusion kurtosis imaging (DKI) based habitat radiomic features with clinical immune-inflammatory biomarkers to predict lymphatic metastatic risk in RC.

**Materials and methods:**

This prospective study included 151 pathologically confirmed patients with rectal adenocarcinoma who underwent preoperative MRI (training cohort: 105 cases; testing cohort: 46 cases). Two radiologists manually delineated the whole-tumor VOI slice by slice on the mean diffusivity (MD) maps using ITK-SNAP software, and the VOIs were subsequently mapped onto the mean kurtosis (MK) maps. K-means clustering was applied for subregion segmentation. Predictive models for LVI and LNM were built using the Random Forest and Extra Trees algorithms, respectively. The Shapley additive explanation method was used to quantify the contribution of each feature to the decision-making of the combined model (Model 3).

**Results:**

Logistic regression analysis demonstrated NHR and EMVI as independent predictors of LVI, while BMI, CA19-9, PNI, and EMVI were independent predictors of LNM. Model 3, which integrated clinical immune-inflammatory biomarkers, conventional radiomic features, and habitat radiomic features, demonstrated the best performance. The AUCs for predicting LVI and LNM were 0.937 vs. 0.864 and 0.901 vs. 0.947 in the training and testing cohorts, respectively.

**Conclusion:**

The habitat radiomics score is a novel and robust quantitative biomarker. Model 3 has demonstrated good performance in assessing the risk of lymphatic metastasis of RC.

**Critical relevance statement:**

Habitat radiomics features derived from DKI parameter maps, combined with clinical immune-inflammatory biomarkers, can predict the risk of lymphatic metastasis of RC, potentially complementing biopsy-based identification of high-risk regions and advancing risk stratification for clinical decision-making in RC management.

**Key Points:**

- Accurate assessment of lymphatic metastasis risk in rectal cancer is crucial for clinical decision-making and personalized treatment optimization.
- Diffusion kurtosis imaging-derived parameters and habitat radiomic features can quantify and characterize intratumoral heterogeneity.
- The combined model provides higher predictive performance for LVI and LNM in rectal cancer.

**Graphical Abstract:**

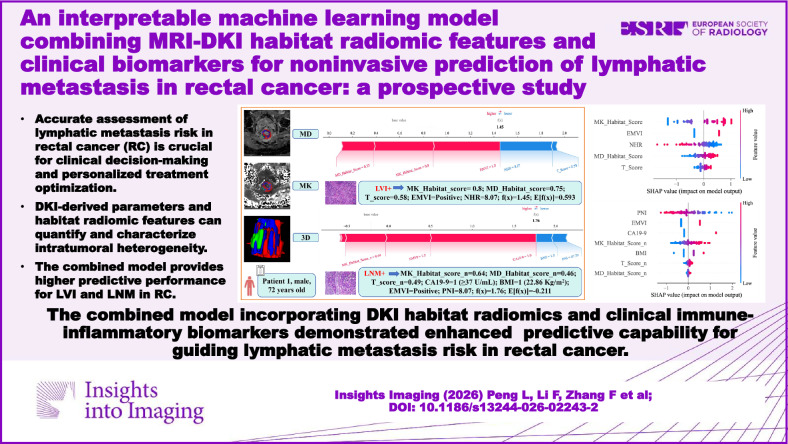

## Introduction

Colorectal cancer (CRC) is a leading cause of digestive system-related cancer deaths and exhibits significant biological heterogeneity [1]. CRC patients with the same TNM stage at initial diagnosis may exhibit significantly different clinical outcomes despite receiving identical treatment regimens. Although the TNM staging system remains a fundamental tool for guiding treatment decisions and evaluating prognosis, growing evidence suggests have emphasized the need to incorporate other critical pathological prognostic factors, such as tumor budding, extramural vascular invasion (EMVI), lymphovascular invasion (LVI), and tumor deposits, to achieve a more comprehensive assessment of tumor biological behavior and recurrence risk [2–4]. In recent years, despite significant advances in cancer screening, genetic testing, and precision therapy, the overall survival rate of patients with rectal cancer (RC) remains relatively low [3, 4].

LVI is defined as the presence of tumor cells in the endodermal lumen or the outward penetration of lymphatic vessel wall destruction, which is the initial manifestation of lymph nodes and other organ metastasis [5, 6]. Previous studies have reported that the LNM rate in LVI-positive (LVI+) patients is as high as 65.7%, compared to 40% in LVI-negative (LVI−) patients [7]. However, once LNM occurs, it is classified as stage III RC, and the treatment strategy is subsequently adjusted [8]. Current guidelines recommend that LVI+ but LNM-negative (LNM−) patients receive similar management to those with LNM-positive (LNM+), including radical resection or adjuvant chemoradiotherapy [3, 4]. In addition, the RC patients with LNM+ who do not undergo lymph node dissection have a significantly increased risk of local recurrence [2]. Conversely, prophylactic lymph node dissection in cases with uncertain LNM status may compromise immune function and elevate surgical trauma-related risks.

At present, key pathological prognostic factors such as LVI and LNM still rely primarily on postoperative histopathological confirmation. However, due to the significant spatial heterogeneity of tumors, conventional histological sampling may suffer from inappropriate region selection or insufficient sample representativeness, thereby compromising diagnostic accuracy. Therefore, there is an urgent need to develop a preoperative non-invasive comprehensive assessment tool with individualized risk stratification capability to fully reflect the tumor microenvironment (TME), enabling precise prediction of lymphatic metastatic risk in RC.

MRI has become a routinely used imaging modality for RC and provides a reliable assessment of key anatomical structures [9]. However, preoperative identification of LNM relying solely on conventional imaging features, such as lymph node size, morphology, marginal features, and enhancement patterns, still faces significant challenges, particularly in detecting micrometastases or tumor deposits, where accuracy remains insufficient [10]. In recent years, radiomics has shown great potential in assessing tumor heterogeneity [11]. Studies have demonstrated that radiomic features derived from the intratumor or peritumoral regions can effectively predict LNM [12] and LVI [13] in RC patients. However, these studies often assume a uniform distribution of features within the volume of interest (VOI), inherently overlooking the biological phenotypic heterogeneity within intratumoral regions [14].

Influenced by local perfusion, cellularity, hypoxia, and immune status, tumors can develop distinct intratumoral subregions with unique biological characteristics and clinical behaviors. More importantly, different subregions may exhibit divergent growth and invasion patterns, as well as heterogeneous responses to therapy and prognosis [15]. In recent years, radiomics approaches based on tumor habitats have demonstrated potential value in RC imaging studies, having been applied to assess MSI-high subregions [16], predict metachronous liver metastasis [17], and LVI [18]. Diffusion kurtosis imaging (DKI), a non-Gaussian diffusion model, provides greater sensitivity to microstructural complexity, and DKI-derived parameters or histogram features have shown promise in evaluating tumor budding [19–21]. However, no studies to date have systematically assessed lymphatic metastatic risk in RC using DKI-based habitat radiomics combined with immune-inflammatory biomarkers.

This study aimed to develop a combined model integrating DKI-based radiomics features with clinical immune-inflammatory markers to predict the risk of lymphatic metastasis in RC. Additionally, the SHAP method is employed for interpretability analysis of the contribution of each feature in the model to evaluate its clinical application value.

## Materials and methods

This prospective study was conducted in accordance with the principles of the Declaration of Helsinki and was approved by the Institutional Review Board (IRB.2022-370).

### Patients

**Fig. 1:**
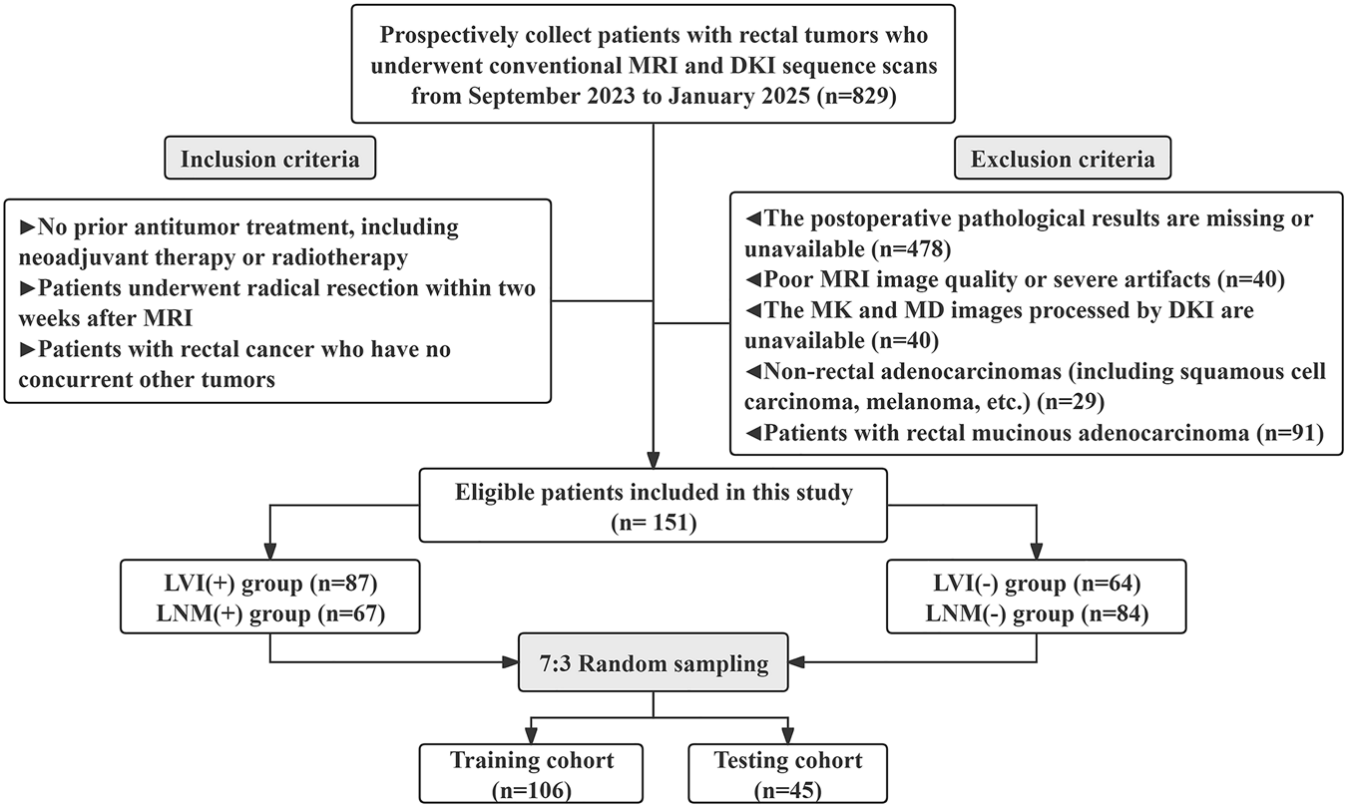
Detailed flowchart of the inclusion and exclusion criteria for the patients in this study

This study prospectively enrolled 829 patients with rectal tumors who underwent conventional MRI and DKI sequence scans at our institution between September 2023 and January 2025. The inclusion criteria were: (1) no prior antitumor treatment; (2) patients who underwent radical resection within 2 weeks after MRI; (3) patients with rectal cancer who had no concurrent other tumors. The exclusion criteria were: (1) the postoperative pathological results are missing or unavailable; (2) poor MRI image quality or severe artifacts; (3) the mean kurtosis (MK) and mean diffusivity (MD) images processed by DKI are unavailable; (4) non-rectal adenocarcinomas; (5) patients with rectal mucinous adenocarcinoma. Finally, 151 RC patients were included in this study. The patients were randomly assigned to training and testing cohorts in a 7:3 ratio. In addition, patients were separated into two temporal cohorts based on the time of enrollment: September 2023 to May 2024 (Time Cohort 1) and May 2024 to January 2025 (Time Cohort 2). The detailed inclusion and exclusion flowchart for RC patients is illustrated in Fig. 1. The methods for collecting baseline clinical data and calculating immune-inflammatory biomarkers are described in Supplementary Material 1 (Appendix E1–E2). LVI and LNM pathological evaluation criteria are described in Supplementary Material 1 (Appendix E3).

### MRI image acquisition and postprocessing of DKI sequences

All patients underwent preoperative 3.0-T MRI, with specific imaging protocols and procedures detailed in Supplementary Material 1 (Appendix E4). DKI images were exported from the PACS system in DICOM format and converted to NIfTI format using MRIcroGL software (https://www.nitrc.org/projects/mricrogl/). The converted images were then imported into the MitkDiffusion workstation (https://github.com/MIC-DKFZ/MITK-Diffusion) for postprocessing, resulting in the generation of MK and MD maps.

### Image preprocessing and segmentation

All the original MRI images have undergone appropriate preprocessing to eliminate heterogeneity among images and improve the repeatability and comparability of the results. Detailed image preprocessing and tumor segmentation methods are provided in Supplementary Material 1 (Appendix E5).

### Tumor habitat region generation

The VOIs were clustered into subregions based on voxel intensity and radiomic feature values extracted from the MD and MK maps. 18 first-order radiomic features were extracted from each voxel within the VOIs on the DKI parametric maps using the gen_roi_rad_features package in the PyCharm Community platform (version 2024.3.5). The specific features are listed in Supplementary Material 2, Fig. S1. Using voxel values and 18 first-order radiomic features, k-means clustering was performed at the population level to segment the VOIs into multiple subregions. Euclidean distances based on voxel intensity and radiomic feature values were used to calculate the similarity between samples, and the number of habitat clusters was tested across a range from 2 to 9. The Calinski-Harabasz index, Silhouette Coefficient, and Davies-Bouldin index were calculated to determine the optimal number of habitat clusters.

### Feature extraction and selection

Radiomics features were independently extracted from different subregions and the entire VOI on both MK and MD maps using PyRadiomics (version 3.0.1). The procedures for radiomic feature selection and dimensionality reduction are provided in Supplementary Material 1 (Appendix E6).

### Construction of conventional and habitat radiomics models

The conventional radiomics score was separately calculated based on the MK and MD maps. To further investigate the influence of the intratumoral microenvironment on the lymphatic metastatic risk in RC, habitat subregions were generated according to the optimal number of clusters. Additionally, radiomic features from these subregions were integrated to construct a comprehensive TME signature. Predictive models for LVI and LNM were developed using the Random Forest and Extra Trees machine learning algorithms, respectively. Additionally, considering that the imbalanced classes would affect the classification performance, we used the synthetic minority oversampling technique to synthesize a virtual balanced training cohort for modeling. Construction of clinical and combined models is described in Supplementary Material 1 (Appendix E7).

### Model performance evaluation

**Fig. 2:**
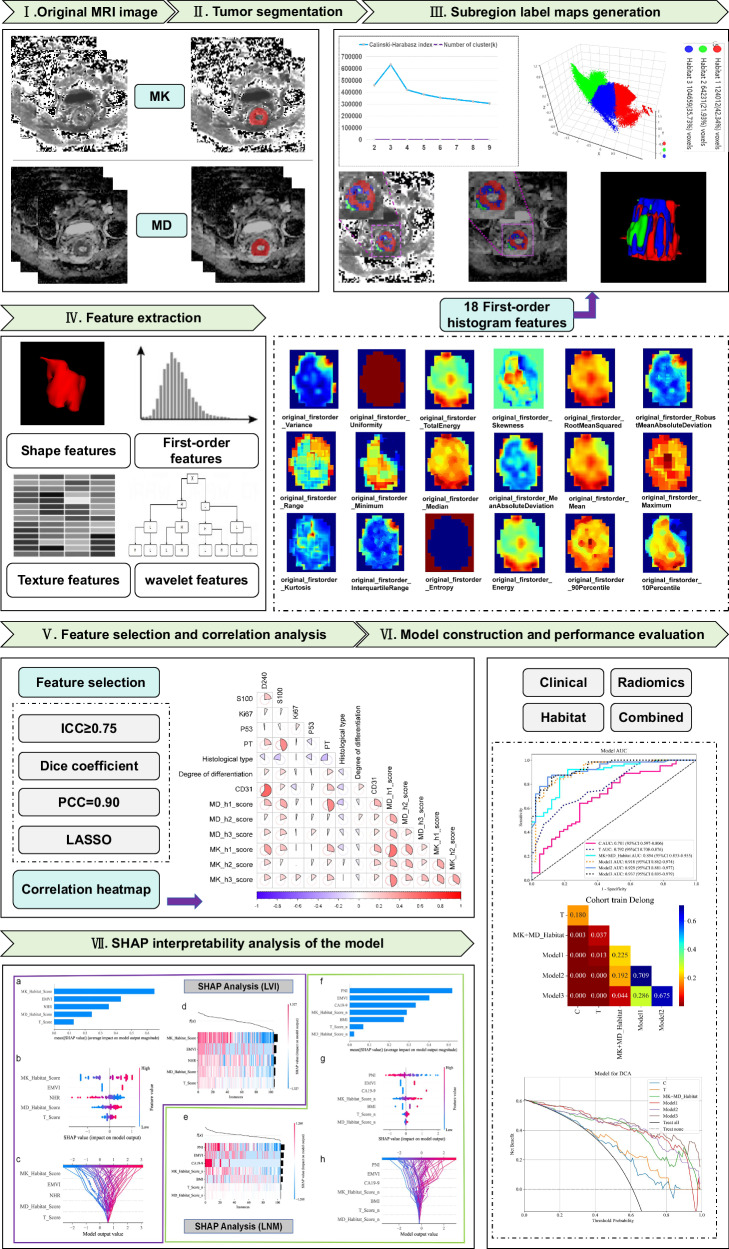
The radiomics research process of this habitat

The predictive performance of the model was evaluated using the receiver operating characteristic (ROC) curve, DeLong test, and integrated discrimination improvement (IDI). The goodness-of-fit of the model was assessed using calibration curves, while the clinical applicability and net benefit were evaluated through decision curve analysis (DCA). Based on the study results, we used SHAP to visualize and analyze the prediction process of the LVI and LNM models. The radiomics research process of this habitat is shown in Fig. 2.

### Statistical analysis

The Kolmogorov–Smirnov test was used to assess the normality of the data distribution. For normally distributed continuous variables, intergroup comparisons were conducted using the independent samples *t*-test. For continuous variables that did not follow a normal distribution, the Mann–Whitney U test was used. Categorical variables were expressed as percentages, and group comparisons for count data were performed using the Chi-square test or Fisher’s exact test. The correlation between radiomics features was analyzed using Spearman correlation analysis. All statistical analyses were performed using Python (version 3.7.3; https://www.python.org/) and R software (version 4.0.4; https://www.r-project.org/). All analyses were two-tailed, and *p* < 0.05 was considered statistically significant.

## Results

### Baseline characteristics of the study cohorts

Table 1 summarizes the clinical baseline characteristics of the patients. A total of 151 patients were enrolled in this study (mean age: 61.88 ± 11.04 years). Between the LVI+ and LVI− groups, significant differences were observed in NHR and EMVI, whereas the remaining indicators demonstrated no statistically significant differences (*p* > 0.05). Between the LNM+ and LNM− groups, significant differences were found in PNI, LHR, HALP, NLR, PLR, MLR, SII, L, EOS, CA19-9, MR-N, and EMVI, whereas the remaining indicators demonstrated no statistically significant differences (*p* > 0.05). Table 2 univariate and multivariate logistic regression analyses demonstrated that NHR and EMVI were independent predictors of LVI, while BMI, CA19-9, PNI, and EMVI were identified as independent predictors of LNM.

**Table 1:** Comparison of clinical baseline characteristics in 151 patients with rectal cancer

Variables	Total (*n* = 151)	LVI− (*n* = 64)	LVI+ (*n* = 87)	*p*-value	LNM− (*n* = 84)	LNM+ (*n* = 67)	*p*-value
Age (years), mean ± SD	61.881 ± 11.036	60.547 ± 10.814	62.862 ± 11.157	0.204	60.500 (56.000, 70.000)	63.000 (53.000, 69.000)	0.475
Sex, *n* (%)				0.634			0.805
Male	103 (68.212)	45 (70.312)	58 (66.667)		58 (69.048)	45 (67.164)	
Female	48 (31.788)	19 (29.688)	29 (33.333)		26 (30.952)	22 (32.836)	
Tumor status, *n* (%)				0.434			0.299
EORC	22 (14.570)	11 (17.188)	11 (12.644)		10 (11.905)	12 (17.910)	
LORC	129 (85.430)	53 (82.812)	76 (87.356)		74 (88.095)	55 (82.090)	
History of colorectal polyps, *n* (%)				0.998			0.692
Yes	92 (60.927)	39 (60.938)	53 (60.920)		50 (59.524)	42 (62.687)	
No	59 (39.073)	25 (39.062)	34 (39.080)		34 (40.476)	25 (37.313)	
BMI (kg/m^2^), *n* (%)				0.829			0.186
< 18.5	7 (4.636)	4 (6.250)	3 (3.448)		5 (5.952)	2 (2.985)	
18.5–24	86 (56.954)	36 (56.250)	50 (57.471)		48 (57.143)	38 (56.716)	
24–28	49 (32.450)	21 (32.812)	28 (32.184)		29 (34.524)	20 (29.851)	
≥ 28	9 (5.960)	3 (4.688)	6 (6.897)		2 (2.381)	7 (10.448)	
PNI, mean ± SD	46.008 ± 6.032	46.654 ± 5.753	45.532 ± 6.219	0.260	47.505 ± 5.935	44.130 ± 5.654	**< 0.001**
NHR, M (Q₁, Q₃)	3.930 (2.775, 5.510)	4.330 (3.053, 6.093)	3.580 (2.555, 4.990)	**0.045**	3.750 (2.732, 5.462)	3.990 (2.855, 6.120)	0.544
MHR, M (Q₁, Q₃)	0.480 (0.350, 0.660)	0.480 (0.395, 0.667)	0.450 (0.325, 0.660)	0.382	0.475 (0.360, 0.620)	0.480 (0.325, 0.715)	0.799
LHR, M (Q₁, Q₃)	1.550 (1.125, 2.205)	1.535 (1.200, 2.135)	1.560 (1.105, 2.205)	0.995	1.790 (1.245, 2.252)	1.300 (1.010, 1.970)	**0.006**
PHR, M (Q₁, Q₃)	212.770 (162.050, 304.065)	216.115 (166.985, 290.767)	210.530 (159.540, 314.425)	0.968	206.715 (165.318, 285.710)	238.100 (155.995, 310.205)	0.295
SIRI, M (Q₁, Q₃)	1.000 (0.625, 1.695)	1.055 (0.725, 1.818)	0.930 (0.560, 1.630)	0.083	0.935 (0.595, 1.290)	1.060 (0.720, 2.020)	0.076
ENLR, M (Q₁, Q₃)	0.070 (0.040, 0.105)	0.065 (0.037, 0.105)	0.070 (0.040, 0.105)	0.872	0.065 (0.040, 0.120)	0.070 (0.040, 0.100)	0.915
HALP, M (Q₁, Q₃)	40.970 (24.075, 56.820)	42.910 (23.655, 56.422)	39.380 (24.150, 56.945)	0.557	47.510 (29.695, 63.475)	28.360 (17.135, 46.245)	**< 0.001**
AISI, M (Q₁, Q₃)	199.900 (109.215, 342.185)	215.135 (126.067, 478.105)	180.880 (100.820, 329.665)	0.195	180.905 (102.055, 318.090)	245.770 (134.915, 469.430)	0.066
NLR, M (Q₁, Q₃)	2.300 (1.620, 3.510)	2.460 (1.740, 3.865)	2.190 (1.495, 3.350)	0.170	2.065 (1.477, 2.925)	2.500 (1.795, 4.085)	**0.019**
PLR, M (Q₁, Q₃)	138.010 (95.945, 178.740)	135.360 (94.875, 184.488)	146.230 (98.020, 174.680)	0.903	110.210 (89.405, 158.702)	154.860 (116.470, 239.110)	**< 0.001**
MLR, M (Q₁, Q₃)	0.280 (0.220, 0.410)	0.300 (0.217, 0.453)	0.280 (0.220, 0.380)	0.554	0.260 (0.200, 0.352)	0.330 (0.260, 0.465)	**< 0.001**
SII, M (Q₁, Q₃)	454.600 (296.530, 794.395)	469.005 (304.040, 788.393)	441.750 (274.300, 768.580)	0.264	392.240 (264.235, 687.375)	531.050 (348.145, 876.685)	**0.015**
MR d, M (Q₁, Q₃)	17.000 (13.250, 20.000)	17.000 (13.875, 20.300)	16.800 (13.000, 19.000)	0.365	16.400 (13.000, 19.000)	17.000 (13.900, 20.000)	0.255
HDL (mmol/L), *n* (%)				0.739			0.481
≤ 1	45 (29.801)	20 (31.250)	25 (28.736)		27 (32.143)	18 (26.866)	
> 1	106 (70.199)	44 (68.750)	62 (71.264)		57 (67.857)	49 (73.134)	
TC (mmol/L), *n* (%)				0.907			0.123
< 5.2	135 (89.404)	57 (89.062)	78 (89.655)		78 (92.857)	57 (85.075)	
≥ 5.2	16 (10.596)	7 (10.938)	9 (10.345)		6 (7.143)	10 (14.925)	
LDL (mmol/L), *n* (%)				1.000			0.460
< 3.4	147 (97.351)	62 (96.875)	85 (97.701)		83 (98.810)	64 (95.522)	
≥ 3.4	4 (2.649)	2 (3.125)	2 (2.299)		1 (1.190)	3 (4.478)	
TG (mmol/L), *n* (%)				0.424			0.419
< 1.7	113 (74.834)	50 (78.125)	63 (72.414)		65 (77.381)	48 (71.642)	
≥ 1.7	38 (25.166)	14 (21.875)	24 (27.586)		19 (22.619)	19 (28.358)	
Blood glucose (mmol/L), *n* (%)				0.207			0.948
3.9–6.1	119 (78.808)	50 (78.125)	69 (79.310)		67 (79.762)	52 (77.612)	
< 3.9	7 (4.636)	1 (1.562)	6 (6.897)		4 (4.762)	3 (4.478)	
> 6.1	25 (16.556)	13 (20.312)	12 (13.793)		13 (15.476)	12 (17.910)	
ALB (g/L), *n* (%)				0.291			0.091
≥ 40	61 (40.397)	29 (45.312)	32 (36.782)		39 (46.429)	22 (32.836)	
< 40	90 (59.603)	35 (54.688)	55 (63.218)		45 (53.571)	45 (67.164)	
PLT, *n* (%)				0.783			0.853
100–300	135 (89.404)	56 (87.500)	79 (90.805)		76 (90.476)	59 (88.060)	
< 100	7 (4.636)	4 (6.250)	3 (3.448)		4 (4.762)	3 (4.478)	
> 300	9 (5.960)	4 (6.250)	5 (5.747)		4 (4.762)	5 (7.463)	
WBC (× 10^9^/L), *n* (%)				0.054			0.828
3.5–9.5	121 (80.132)	49 (76.562)	72 (82.759)		66 (78.571)	55 (82.090)	
< 3.5	14 (9.272)	4 (6.250)	10 (11.494)		8 (9.524)	6 (8.955)	
> 9.5	16 (10.596)	11 (17.188)	5 (5.747)		10 (11.905)	6 (8.955)	
HB (g/L), *n* (%)				0.206			0.286
120–160	97 (64.238)	46 (71.875)	51 (58.621)		58 (69.048)	39 (58.209)	
< 120	36 (23.841)	11 (17.188)	25 (28.736)		16 (19.048)	20 (29.851)	
> 160	18 (11.921)	7 (10.938)	11 (12.644)		10 (11.905)	8 (11.940)	
RBC (× 10^12^/L), *n* (%)				0.549			0.682
4.0–5.5	113 (74.834)	49 (76.562)	64 (73.563)		63 (75.000)	50 (74.627)	
< 4.0	31 (20.530)	11 (17.188)	20 (22.989)		16 (19.048)	15 (22.388)	
> 5.5	7 (4.636)	4 (6.250)	3 (3.448)		5 (5.952)	2 (2.985)	
N (× 10^9^/L), *n* (%)				0.059			0.552
1.2–7.0	134 (88.742)	53 (82.812)	81 (93.103)		76 (90.476)	58 (86.567)	
< 1.2	3 (1.987)	1 (1.562)	2 (2.299)		2 (2.381)	1 (1.493)	
> 7.0	14 (9.272)	10 (15.625)	4 (4.598)		6 (7.143)	8 (11.940)	
L (× 10^9^/L), *n* (%)				0.894			**0.027**
1.2–3.5	100 (66.225)	42 (65.625)	58 (66.667)		62 (73.810)	38 (56.716)	
< 1.2	51 (33.775)	22 (34.375)	29 (33.333)		22 (26.190)	29 (43.284)	
M (× 10^9^/L), *n* (%)				0.156			1.000
0.1–1.0	147 (97.351)	63 (98.438)	84 (96.552)		81 (96.429)	66 (98.507)	
< 0.1	1 (0.662)	1 (1.562)	0 (0.00)		1 (1.190)	0 (0.00)	
> 1.0	3 (1.987)	0 (0.00)	3 (3.448)		2 (2.381)	1 (1.493)	
EOS (× 10^9^/L), *n* (%)				0.799			**0.044**
0.05–0.5	121 (80.132)	50 (78.125)	71 (81.609)		72 (85.714)	49 (73.134)	
< 0.05	27 (17.881)	13 (20.312)	14 (16.092)		12 (14.286)	15 (22.388)	
> 0.5	3 (1.987)	1 (1.562)	2 (2.299)		0 (0.00)	3 (4.478)	
CEA (ng/mL), *n* (%)				0.921			0.312
< 5	88 (58.278)	37 (57.812)	51 (58.621)		52 (61.905)	36 (53.731)	
≥ 5	63 (41.722)	27 (42.188)	36 (41.379)		32 (38.095)	31 (46.269)	
CA19-9 (U/mL), *n* (%)				0.601			**< 0.001**
< 37	132 (87.417)	57 (89.062)	75 (86.207)		82 (97.619)	50 (74.627)	
≥ 37	19 (12.583)	7 (10.938)	12 (13.793)		2 (2.381)	17 (25.373)	
CA7-24 (U/mL), *n* (%)				0.408			0.309
< 6.9	133 (88.079)	58 (90.625)	75 (86.207)		76 (90.476)	57 (85.075)	
≥ 6.9	18 (11.921)	6 (9.375)	12 (13.793)		8 (9.524)	10 (14.925)	
Tumor location, *n* (%)				0.580			0.325
Upper	37 (24.503)	13 (20.312)	24 (27.586)		18 (21.429)	19 (28.358)	
Middle	57 (37.748)	26 (40.625)	31 (35.632)		30 (35.714)	27 (40.299)	
Lower	57 (37.748)	25 (39.062)	32 (36.782)		36 (42.857)	21 (31.343)	
MR-T, *n* (%)				0.082			0.418
T1	1 (0.662)	1 (1.562)	0 (0.00)		1 (1.190)	0 (0.00)	
T2	33 (21.854)	13 (20.312)	20 (22.989)		20 (23.810)	13 (19.403)	
T3	104 (68.874)	48 (75.000)	56 (64.368)		58 (69.048)	46 (68.657)	
T4	13 (8.609)	2 (3.125)	11 (12.644)		5 (5.952)	8 (11.940)	
MR-N, *n* (%)				0.399			**0.009**
N0	36 (23.841)	18 (28.125)	18 (20.690)		26 (30.952)	10 (14.925)	
N1	47 (31.126)	21 (32.812)	26 (29.885)		29 (34.524)	18 (26.866)	
N2	68 (45.033)	25 (39.062)	43 (49.425)		29 (34.524)	39 (58.209)	
MRF, *n* (%)				0.302			0.262
Negative	119 (78.808)	53 (82.812)	66 (75.862)		69 (82.143)	50 (74.627)	
Positive	32 (21.192)	11 (17.188)	21 (24.138)		15 (17.857)	17 (25.373)	
EMVI, *n* (%)				**< 0.001**			**0.002**
Negative	97 (64.238)	52 (81.250)	45 (51.724)		63 (75.000)	34 (50.746)	
Positive	54 (35.762)	12 (18.750)	42 (48.276)		21 (25.000)	33 (49.254)	

Data are the number of patients, and data in parentheses are percentages. There were no missing values in the patients’ baseline clinical data

*EORC* early-onset rectal cancer, *LORC* late-onset rectal cancer, *CA19-9* carbohydrate antigen 19-9, *CA7-24* carbohydrate antigen 7-24, *CEA* carcinoembryonic antigen, *BMI* body mass index, *NHR* neutrophils to high-density lipoprotein ratio, *MHR* monocytes to high-density lipoprotein ratio, *LHR* lymphocytes to high-density lipoprotein ratio, *PHR* platelets to high-density lipoprotein ratio, *M* monocyte, *L* lymphocyte, *PLT* platelet, *MPV* mean platelet volume, *N* neutrophils, *M* monocytes, *WBC* white blood cell, *TC* total cholesterol, *TG* triglycerides, *HDL* high-density lipoprotein, *LDL* low density lipoprotein, *HB* hemoglobin, *RBC* red blood cell, *SIRI* inflammation response index, *EOS* eosinophil count, *ALB* albumin, *ENLR* eosinophil count to lymphocytes ratio, *AISI* aggregate inflammation systemic index, *NLR* neutrophil to lymphocyte ratio, *PLR* platelet to lymphocyte ratio, *MLR* monocyte to lymphocytes ratio, *SII* systemic immune-inflammation index, *PNI* prognostic nutritional index, *HALP* (hemoglobin, albumin, lymphocyte, platelet), *MRF* mesorectal fascia, *EMVI* extramural vascular invasion, *MR_d* MRI reported the maximum thickness of the tumor, *MR-T* MRI-reported T stage, *MR-N* MRI-reported N stage.

The bold values indicate *p* < 0.05, representing statistically significant differences between the two groups.

**Table 2:** Logistic regression analysis selected the optimal risk factors for preoperative prediction of LVI and LNM for rectal cancer

Research on predicting LVI in rectal cancer						
Variables	Univariate logistic regression analysis			Multivariate logistic regression analysis		
	OR	95% CI	*p*-value	OR	95% CI	*p*-value
WBC (> 9.5 × 10^9^/L vs. 3.5–9.5 × 10^9^/L)	0.309	0.101–0.946	0.040	–	–	–
HB (< 120 g/L vs. 120–160 g/L)	2.050	0.909–4.624	0.084	–	–	–
N (> 7.0 × 10^9^/L vs. 1.2–7.0 × 10^9^/L)	0.262	0.078–0.878	0.030	–	–	–
NHR	0.888	0.791–0.998	0.046	0.864	0.761–0.982	0.025^*^
SIRI	0.887	0.771–1.020	0.093	–	–	–
AISI	0.999	0.999–1.000	0.045	–	–	–
SII	0.999	0.999–1.000	0.040	–	–	–
EMVI (positive vs. negative)	4.044	1.900–8.610	< 0.001	4.209	1.927–9.194	< 0.001^***^

Research on predicting LNM in rectal cancer						
Variables	Univariate logistic regression analysis			Multivariate logistic regression analysis		
	OR	95% CI	*p*-value	OR	95% CI	*p*-value
BMI (≥ 28 kg/m^2^ vs. < 18.5 kg/m^2^)	8.750	0.903–84.800	0.061	47.682	2.636–862.548	0.009**
ALB (< 40 g/L vs. ≥ 40 g/L)	1.773	0.911–3.451	0.092	–	–	–
L (< 1.2 × 10^9^/L vs. 1.2–3.5 × 10^9^/L)	2.151	1.083–4.269	0.029	–	–	–
CA19-9 (≥ 37 U/mL vs. < 37 U/mL)	13.940	3.090–62.896	0.001	12.388	2.272–67.545	0.004**
MR-N (N2 vs. N0)	3.497	1.460–8.375	0.005	–	–	–
EMVI (positive vs. negative)	2.912	1.463–5.793	0.002	4.138	1.801–9.511	0.001**
LHR	0.536	0.341–0.840	0.007	–	–	–
HALP	0.969	0.953–0.985	< 0.001	–	–	–
PNI	0.904	0.852–0.960	0.001	0.925	0.857–0.999	0.047*
AISI	1.001	1.000–1.001	0.063	–	–	–
NLR	1.098	1.009–1.195	0.030	–	–	–
PLR	1.007	1.003–1.011	0.001	–	–	–
MLR	5.443	1.411–20.995	0.014	–	–	–

*95% CI* 95% confidence interval, *OR* odds ratio, *WBC* white blood cell, *HB* hemoglobin, *N* neutrophil, *NLR* neutrophil to lymphocyte ratio, *SIRI* inflammation response index, *AISI* aggregate inflammation systemic index, *SII* systemic immune-inflammation index, *EMVI* MRI-reported EMVI, *BMI* body mass index, *ALB* albumin, *L* lymphocyte, *CA19-9* carbohydrate antigen 19-9, *MR-N* MRI-reported N stage, *LHR* lymphocytes to high-density lipoprotein ratio, *PNI* prognostic nutritional index, *PLR* platelet to lymphocyte ratio, *MLR* monocyte to lymphocyte ratio, *HALP* (hemoglobin, albumin, lymphocyte, platelet)

* *p* < 0.05, ** *p* < 0.01, *** *p* < 0.001. The bold values represent independent predictive factors after multivariate logistic regression analysis.

### Subregion clustering, radiomics feature extraction and selection

The VOI segmentation and conventional radiomic features demonstrated excellent consistency and robustness (all ICCs > 0.9, with a mean Dice coefficient of 0.899), as detailed in Supplementary Material 2, Table S1 and Fig. S2. The Calinski-Harabasz index, Silhouette Coefficient, and Davies-Bouldin index identified three as the optimal number of clusters (Supplementary Material 2, Fig. S3a–c). The voxel count and corresponding proportion for each subregion are shown in Supplementary Material 2, Fig. S3d. Habitat 1 accounted for the largest proportion (42.34%). In the LVI prediction study, 10 and 7 subregion radiomics features were selected from the MD and MK maps, respectively, along with 4 conventional features jointly derived from MD + MK maps. For LNM prediction, 13 and 15 subregion radiomics features were selected from the MD and MK maps, respectively, and 5 conventional features were jointly selected from MD + MK maps. The coefficient distribution of selected features for each model is illustrated in Supplementary Material 2, Fig. S4. The permutation test results demonstrated that the final selected modeling features exhibited good robustness (Supplementary Material 2, Fig. S5). The results of correlation analysis between the subregion radiomics signatures and pathological features are provided in Supplementary Material 2, Fig. S6.

### Performance comparison of different models and SHAP analysis

**Fig. 3:**
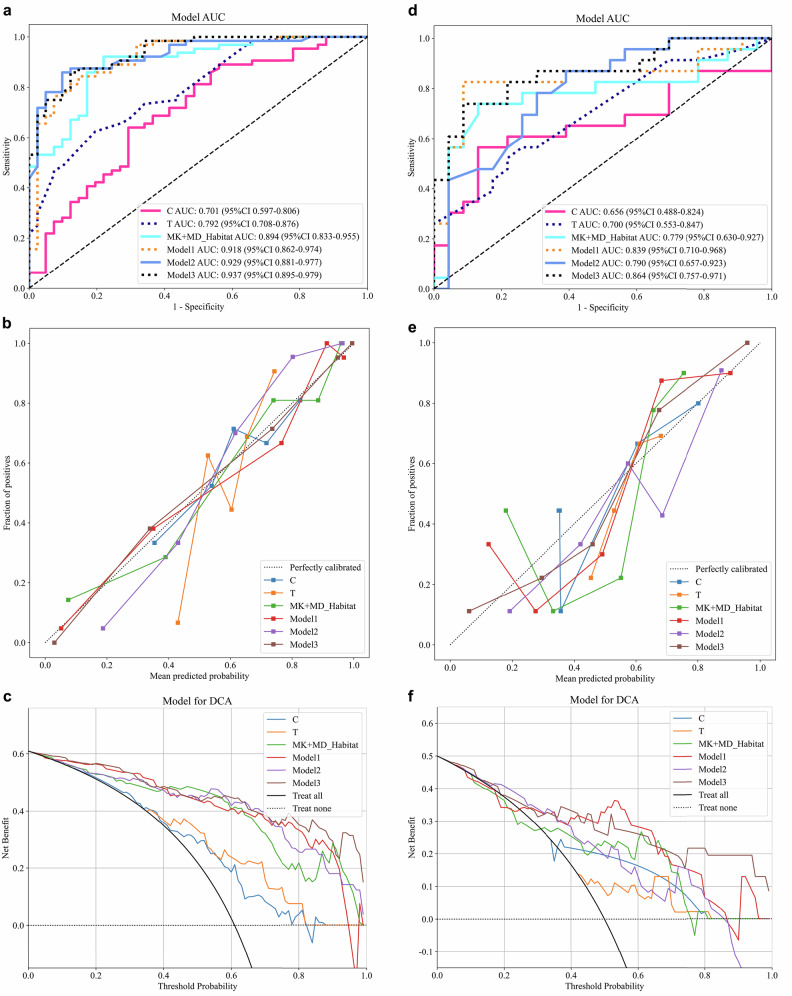
Performance evaluation of different models for predicting LVI. **a**–**c** show the ROC curve, calibration curve, and decision curve for the training cohort, respectively; **d**–**f** show the ROC curve, calibration curve, and decision curve for the testing cohort, respectively

**Fig. 4:**
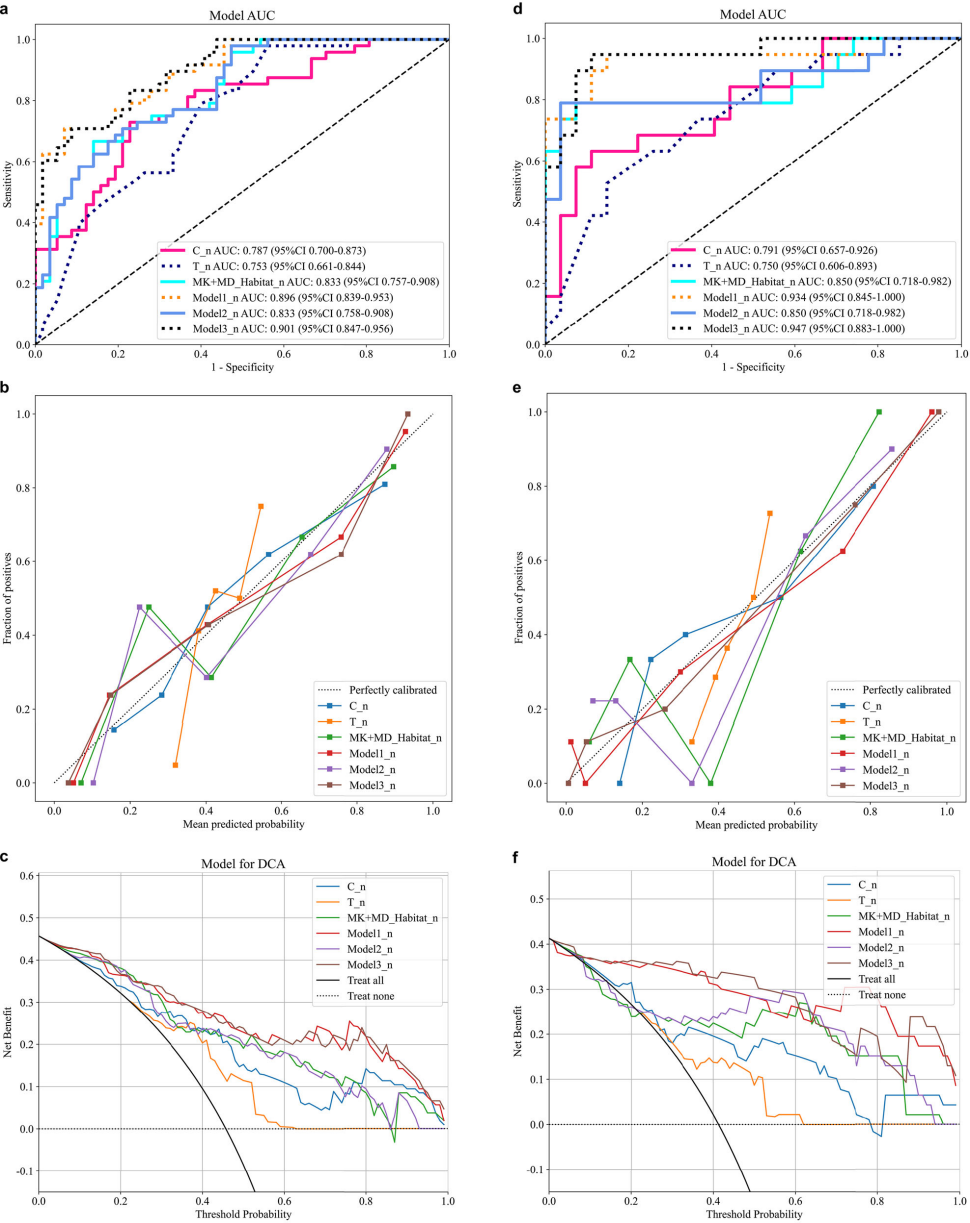
Performance evaluation of different models for predicting LNM. **a**–**c** show the ROC curve, calibration curve, and decision curve for the training cohort, respectively; **d**–**f** show the ROC curve, calibration curve, and decision curve for the testing cohort, respectively

**Fig. 5:**
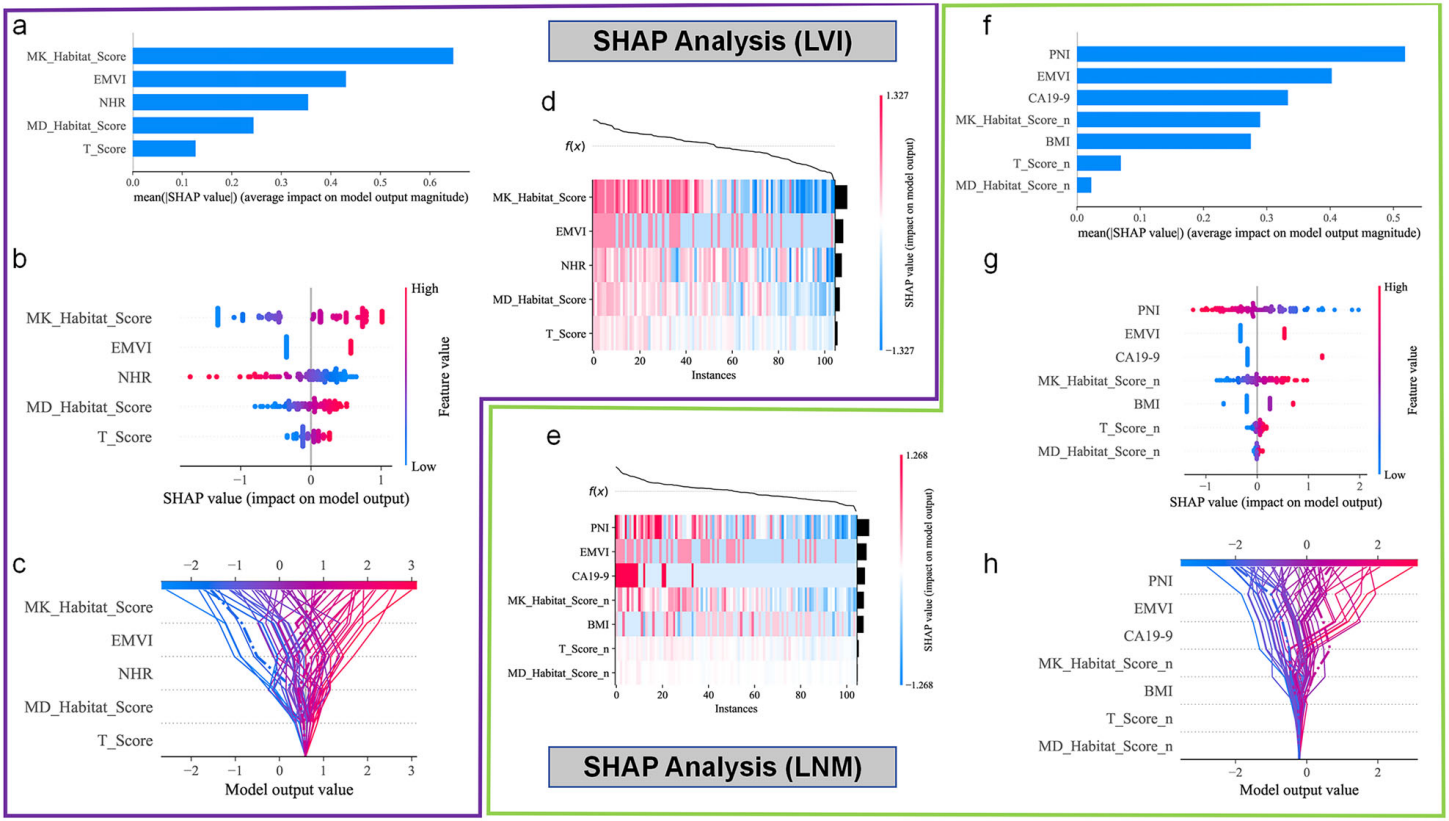
SHAP interpretability analysis. **a**–**d** and **e**–**h** are the SHAP analysis visualizations of Model 3 for predicting LVI and LNM, respectively; **a** and **f** are bar plots of feature importance; **b** and **g** are summary plots; **c** and **h** are decision plots; **d** and **e** are heatmaps

**Fig. 6:**
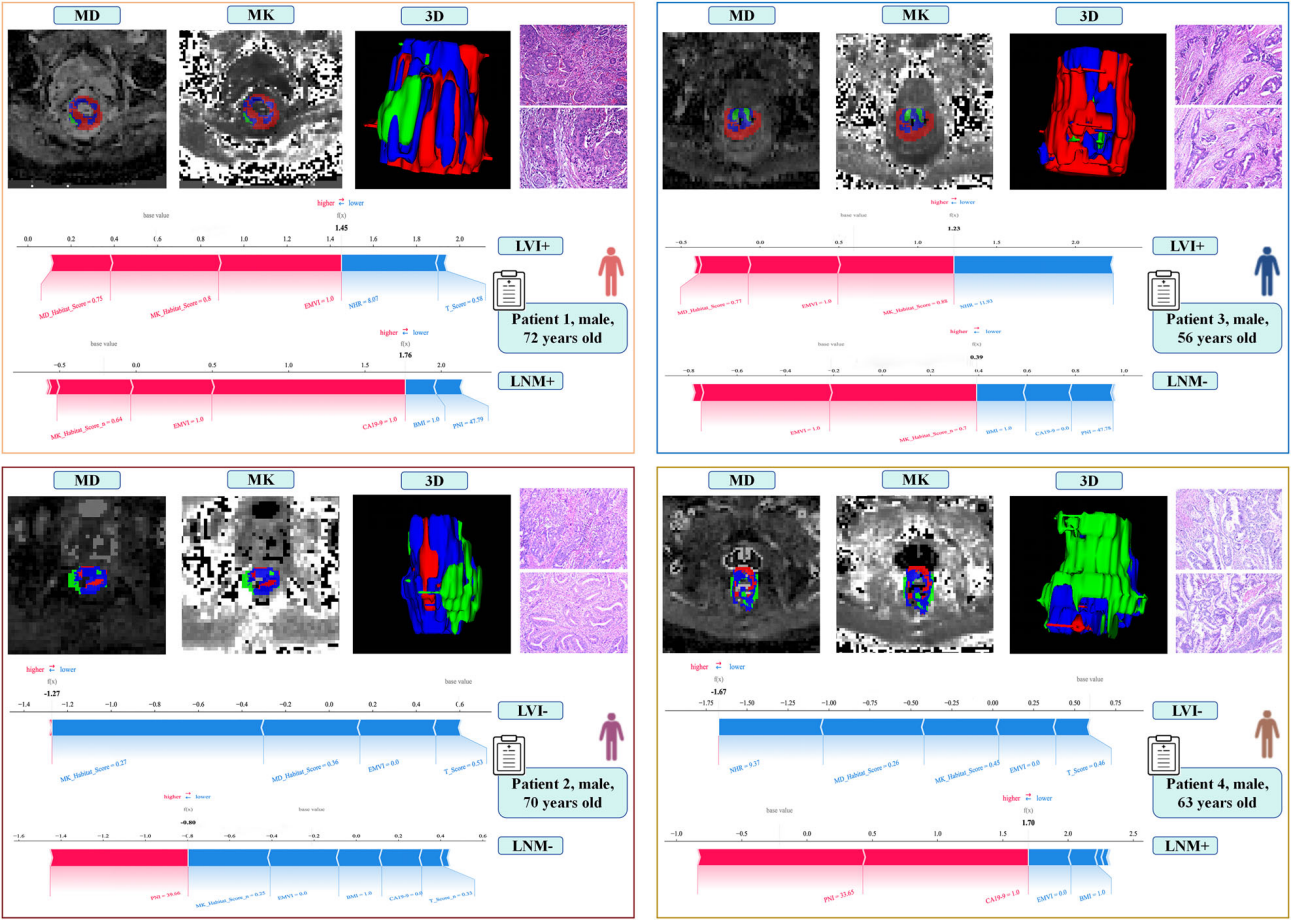
Case analysis. This figure shows the SHAP-based clinical decision analysis of Model 3 for Patient 1 (LVI+ and LNM+), Patient 2 (LVI- and LNM−), Patient 3 (LVI+ and LNM−), and Patient 4 (LVI− and LNM+), respectively

ROC analysis demonstrated that the CA199_CEA_TN model performed well in the clinical baseline model (Supplementary Material 2, Fig. S7). Model 1 and Model 3 demonstrated comparable performance in predicting the risk of lymphatic metastasis. However, considering the robustness and generalizability of the models across both prognostic outcomes, as well as the complementary value of different types of features in representing the biological characteristics of tumors, we ultimately selected Model 3 as the optimal predictive model in this study. For LVI prediction, Model 3 yielded AUCs of 0.937 and 0.864 in the training and testing cohorts, respectively; for LNM prediction, AUCs were 0.901 and 0.947 in the training and testing cohorts, respectively (Figs. 3, 4 and Table 3). The calibration curves showed good agreement with the ideal reference line (Figs. 3b, e, 4b, e). DCA demonstrated that Model 3 provided greater net benefit (Figs. 3c, f, 4c, f). In the LVI prediction task, the AUC values of Model 3 were 0.896 and 0.945 in Time Cohort 1 and Time Cohort 2, respectively. For LNM prediction, the AUC values were 0.916 and 0.910 in the two temporal cohorts. Regarding clinical net benefit assessed by DCA, the decision threshold ranges for Model 3 were 0.04–0.80 for LVI prediction and 0.01–0.93 for LNM prediction in Time Cohort 1; and 0.04–0.92 and 0.03–0.86, respectively, in Time Cohort 2. The corresponding ROC and DCA curves are presented in Supplementary Material 2, Fig. S8, and the model performance comparison is summarized in Supplementary Material 2, Table S2. In both cohorts, MK_Habitat_Score, MD_Habitat_Score, and T_Score were significantly higher in the LVI+ group than in the LVI− group (all *p* < 0.01); similarly, these scores were significantly higher in the LNM+ group than in the LNM− group (Supplementary Material 2, Fig. S9). To enhance interpretability, both global and local SHAP values were calculated for Model 3. Figure 5a–d, e–h presents the global SHAP visualizations for predicting LVI and LNM, respectively. In the individual visualization, Fig. 6 presents four representative examples of RC cases with predicted lymphatic metastatic risk. Details of the SHAP-based interpretability analysis are provided in Supplementary Material 1 (Appendix E8, E9).

**Table 3:** Comparison of ROC performance of different models

Research on predicting LVI in rectal cancer												
Cohort	Model	AUC	95% CI	Accuracy	Sensitivity	Specificity	PPV	NPV	Precision	Recall	F1-score	Threshold
Train	C	0.701	0.597–0.806	0.667	0.641	0.707	0.774	0.558	0.774	0.641	0.701	0.612
Train	MD_Habitat	0.825	0.740–0.910	0.800	0.875	0.683	0.812	0.778	0.812	0.875	0.842	0.534
Train	MK_Habitat	0.853	0.777–0.929	0.810	0.812	0.805	0.867	0.733	0.867	0.812	0.839	0.663
Train	MK + MD_Habitat	0.894	0.833–0.955	0.867	0.922	0.780	0.868	0.865	0.868	0.922	0.894	0.577
Train	T	0.792	0.708–0.876	0.695	0.625	0.805	0.833	0.579	0.833	0.625	0.714	0.647
Train	T + C	0.826	0.748–0.904	0.752	0.719	0.805	0.852	0.647	0.852	0.719	0.780	0.645
Train	Model1	0.918	0.862–0.974	0.848	0.844	0.854	0.900	0.778	0.900	0.844	0.871	0.694
Train	Model2	0.929	0.881–0.977	0.876	0.859	0.902	0.932	0.804	0.932	0.859	0.894	0.561
Train	Model3	0.937	0.895–0.979	0.867	0.859	0.878	0.917	0.800	0.917	0.859	0.887	0.624
Test	C	0.656	0.488–0.824	0.717	0.565	0.870	0.812	0.667	0.812	0.565	0.667	0.371
Test	MD_Habitat	0.653	0.489–0.817	0.696	0.522	0.870	0.800	0.645	0.800	0.522	0.632	0.721
Test	MK_Habitat	0.778	0.636–0.920	0.761	0.609	0.913	0.875	0.700	0.875	0.609	0.718	0.747
Test	MK + MD_Habitat	0.779	0.630–0.928	0.804	0.739	0.870	0.850	0.769	0.850	0.739	0.791	0.613
Test	T	0.700	0.553–0.848	0.652	0.522	0.783	0.706	0.621	0.706	0.522	0.600	0.597
Test	T + C	0.813	0.693–0.933	0.739	0.783	0.696	0.720	0.762	0.720	0.783	0.750	0.422
Test	Model1	0.839	0.710–0.968	0.870	0.826	0.913	0.905	0.840	0.905	0.826	0.864	0.549
Test	Model2	0.790	0.657–0.923	0.739	0.783	0.696	0.720	0.762	0.720	0.783	0.750	0.542
Test	Model3	0.864	0.757–0.971	0.826	0.739	0.913	0.895	0.778	0.895	0.739	0.810	0.520

Research on predicting LNM in rectal cancer												
Cohort	Model	AUC	95% CI	Accuracy	Sensitivity	Specificity	PPV	NPV	Precision	Recall	F1-score	Threshold
Train	C_n	0.787	0.700–0.873	0.752	0.729	0.772	0.729	0.772	0.729	0.729	0.729	0.440
Train	MD_Habitat_n	0.759	0.670–0.848	0.705	0.646	0.754	0.689	0.717	0.689	0.646	0.667	0.435
Train	MK_Habitat_n	0.818	0.739–0.898	0.743	0.875	0.632	0.667	0.857	0.667	0.875	0.757	0.386
Train	MK + MD_Habitat_n	0.833	0.757–0.908	0.771	0.667	0.860	0.800	0.754	0.800	0.667	0.727	0.530
Train	T_n	0.753	0.661–0.844	0.686	0.979	0.439	0.595	0.962	0.595	0.979	0.740	0.380
Train	T + C_n	0.843	0.770–0.917	0.771	0.854	0.702	0.707	0.851	0.707	0.854	0.774	0.340
Train	Model1_n	0.896	0.839–0.953	0.829	0.708	0.930	0.895	0.791	0.895	0.708	0.791	0.718
Train	Model2_n	0.833	0.758–0.908	0.733	0.979	0.526	0.635	0.968	0.635	0.979	0.770	0.218
Train	Model3_n	0.901	0.847–0.956	0.819	0.708	0.912	0.872	0.788	0.872	0.708	0.782	0.682
Test	C_n	0.791	0.657–0.926	0.783	0.632	0.889	0.800	0.774	0.800	0.632	0.706	0.493
Test	MD_Habitat_n	0.718	0.563–0.873	0.674	0.684	0.667	0.591	0.750	0.591	0.684	0.634	0.435
Test	MK_Habitat_n	0.846	0.715–0.977	0.870	0.789	0.926	0.882	0.862	0.882	0.789	0.833	0.552
Test	MK + MD_Habitat_n	0.850	0.718–0.982	0.870	0.789	0.926	0.882	0.862	0.882	0.789	0.833	0.544
Test	T_n	0.750	0.606–0.894	0.717	0.526	0.852	0.714	0.719	0.714	0.526	0.606	0.493
Test	T + C_n	0.885	0.790–0.980	0.848	0.947	0.778	0.750	0.955	0.750	0.947	0.837	0.350
Test	Model1_n	0.934	0.846–1.000	0.891	0.947	0.852	0.818	0.958	0.818	0.947	0.878	0.390
Test	Model2_n	0.850	0.718–0.982	0.891	0.789	0.963	0.937	0.867	0.937	0.789	0.857	0.621
Test	Model3_n	0.947	0.883–1.000	0.913	0.947	0.889	0.857	0.960	0.857	0.947	0.900	0.350

C: clinical model; T: MK + MD traditional radiomics model; T + C: MK + MD traditional radiomics model + clinical model; Model1: MK + MD_Habitat + C; Model2: MK + MD_Habitat + T; Model3: MK + MD_Habitat + C + T

*95% CI* 95% confidence interval, *AUC* area under the curve, *PPV* positive predictive value, *NPV* negative predictive value

## Discussion

In this prospective study, we developed habitat-based and conventional radiomics models derived from DKI parameters and constructed a combined model integrating clinical immune-inflammatory biomarkers to noninvasively predict lymphatic metastatic risk in RC. Furthermore, Model 3 provides individualized probabilities for LVI and LNM. Within a multidisciplinary team (MDT) framework, such probability outputs may serve as adjunctive decision-support information, helping to identify patients at higher risk of lymphatic metastasis and to inform personalized treatment planning. In the training cohort, probability thresholds of 0.624 for LVI and 0.682 for LNM, derived using the Youden index, achieved a reasonable balance between sensitivity and specificity. As a conceptual example, patients exceeding these thresholds could be prioritized for MDT discussion regarding neoadjuvant treatment strategies, careful assessment of circumferential resection margin (CRM) safety, and optimization of lymph node dissection. It is worth noting that these thresholds are exploratory and require validation in future multicenter prospective studies.

Conventional single-parameter imaging modalities are limited in their ability to comprehensively characterize the complexity of the tumor microenvironment. Therefore, in this study, we employed more sensitive DKI-derived maps for habitat radiomics analysis. MD can accurately reflect the diffusion characteristics of water molecules within tumor tissues, while MK quantifies the degree to which water molecule diffusion deviates from a Gaussian distribution [22, 23]. Previous studies have shown that elevated MK is typically associated with higher tumor cell density, increased nuclear atypia, and active angiogenesis, whereas reduced MD generally indicates tighter tumor cell arrangement, more restricted extracellular space, and a greater likelihood of metastasis [19, 20, 24, 25]. These findings are consistent with the results of our study. In our comparison of predictive performance for LVI and LNM, both habitat and conventional radiomic features extracted from MK maps outperformed those derived from MD maps. Further pathological correlation analyses revealed that, in LVI prediction, Subregion 1 exhibited the strongest positive correlations with D2-40, CD31, S100, and pathological T stage, suggesting that this subregion may correspond to tumor areas characterized by active lymphangiogenesis, angiogenesis, and stromal invasion. In contrast, for LNM prediction, Subregion 3 showed the strongest positive associations with these markers, indicating a more aggressive tumor microenvironment with a higher propensity for lymphatic spread. These findings demonstrated that the three habitat subregions possess distinct biological significance, reflecting heterogeneous invasive and metastatic “ecological niches” within the tumor microenvironment. Such biologically meaningful habitat partitioning not only strengthens the interpretability of the model but may also provide valuable guidance for surgical planning and targeted pathological sampling in RC.

In the radiomics feature analysis, two features consistently demonstrated stable contributions to both the LVI and LNM prediction models: MD_wavelet_LHL_glszm_SmallAreaEmphasis_h3 and MK_wavelet_LLH_glcm_Imc1. The SmallAreaEmphasis, derived from the gray-level size zone matrix, quantifies the predominance of small, high-intensity regions within the image. Higher values indicate densely clustered signal foci, potentially reflecting microstructural traits of tumor aggressiveness such as high cellular density, uneven cell distribution, or necrotic margins. This feature originated from a highly variable subregion of the MD map, underscoring the link between intratumoral heterogeneity in restricted diffusion areas and invasive potential. The second feature, Imc1, derived from the gray-level co-occurrence matrix, measures nonlinear dependencies of gray-level intensities. Lower Imc1 values denote more disorganized texture patterns, indicative of increased structural heterogeneity. Extracted from the MK map, this feature reflects deviations of water diffusion from Gaussian behavior at the whole-tumor level, typically observed in tumors with highly complex architectures or disrupted microenvironments.

In this study, we found that Model 1 demonstrated slightly better performance than Model 2 in predicting both LVI and LNM. This finding suggests that systemic biological indicators may provide stronger explanatory power for lymphatic metastasis risk compared to tumoral radiomic features alone. Model 3 achieved favorable overall performance in predicting LVI and LNM. These findings underscore the complementary value of systemic biological markers and intratumoral imaging features, indicating that integrating multi-dimensional features not only enables a more comprehensive characterization of tumor heterogeneity and overcomes the limitations of traditional lymph node assessment based solely on size and morphology, but also markedly enhances the predictive performance for LNM and LVI, thereby offering substantial clinical utility for preoperative risk stratification, surgical planning, and individualized treatment decision-making.

In the analysis of clinical features, EMVI emerged as a common independent risk factor for both LVI and LNM. EMVI refers to the direct invasion of venous structures beyond the muscularis propria by the tumor, which is typically assessed on MRI images. With tumor progression, the incidence of LVI and EMVI increases, reflecting enhanced vascular invasion and metastatic potential [26]. The multivariate analysis revealed that the NHR and PNI as independent protective factors for LVI and LNM, respectively (OR < 1), whereas BMI and CA19-9 were independent risk factors for LNM (OR > 1). Previous studies have demonstrated that neutrophils play a dual role in tumor initiation and progression [27]. Our study found that patients in the LVI− group exhibited a higher neutrophil count and elevated NHR, which may be associated with neutrophil-mediated antitumor effects. Malignant tumors are often accompanied by a decline in nutritional status, potentially due to tumor-induced inflammatory responses that suppress albumin synthesis and promote its degradation. This deterioration can negatively affect both nutritional and immune functions, ultimately impacting patient prognosis [28]. Low serum albumin levels may impair DNA replication and antioxidant defenses, while its degradation products can serve as nutrient sources that facilitate tumor proliferation and metastasis [29]. As a tumor marker, elevated levels of CA19-9 have been shown to reflect higher tumor aggressiveness and greater metastatic potential, and are associated with poor prognosis in RC [30, 31]. Additionally, BMI reflects fat reserves and metabolic status. Obesity may promote tumorigenesis and metastasis by activating cell growth pathways; altered glucose metabolism and increased insulin secretion are potential mechanisms linking obesity to a heightened risk of CRC [32].

In addition, this study had some limitations. First, as an exploratory single-center investigation applying the novel DKI technique to assess lymphatic metastatic risk in RC, the sample size was relatively small, the probability threshold of the model was derived based on data from a single center, and the generalizability of the model remains to be validated. Future research should incorporate larger, multicenter, prospective datasets to confirm our findings. Second, subregion segmentation and construction were based on MK and MD pixel values combined with more stable first-order histogram features. Future studies should explore incorporating additional meaningful and robust features into habitat construction to enhance the biological relevance of imaging analysis. Finally, histopathologic images were not used to substantiate observations regarding habitat imaging, as used to validate findings in an earlier study of habitat imaging [33]. In future work, we plan to perform validation using whole-tumor gross pathology and immunohistochemistry in RC.

## Conclusions

Microenvironmental imaging techniques that integrate tumor imaging characteristics can help predict the lymphatic metastatic risk in RC. Incorporating information from tumor subregions, clinical immune-inflammatory biomarkers, and whole-tumor characteristics holds the potential to enhance predictive performance.

## Supplementary information

Supplementary information

## Data Availability

The data used and analyzed during the current study are available from the corresponding authors on reasonable request.

## Abbreviations

ALB: Albumin
AUC: Area under the curve
BMI: Body mass index
CA19-9: Carbohydrate antigen 19-9
CEA: Carcinoembryonic antigen
CRC: Colorectal cancer
DCA: Decision curve analysis
EMVI: Extramural venous invasion
L: Lymphocyte
LNM: Lymph node metastasis
LVI: Lymphovascular invasion
M: Monocyte
MRI: Magnetic resonance imaging
N: Neutrophils
RC: Rectal cancer
SHAP: Shapley additive explanation

## References

[1] Bray F, Laversanne M, Sung H et al (2024) Global cancer statistics 2022: GLOBOCAN estimates of incidence and mortality worldwide for 36 cancers in 185 countries. CA Cancer J Clin 74:229–263 38572751 10.3322/caac.21834

[2] Cervantes A, Adam R, Roselló S et al (2023) Metastatic colorectal cancer: ESMO clinical practice guideline for diagnosis, treatment and follow-up. Ann Oncol 34:10–32 36307056 10.1016/j.annonc.2022.10.003

[3] Benson AB, Venook AP, Al-Hawary MM et al (2022) Rectal cancer, version 2.2022, NCCN clinical practice guidelines in oncology. J Natl Compr Canc Netw 20:1139–1167 36240850 10.6004/jnccn.2022.0051

[4] Chen L, Hu H, Yuan Y, Weng S (2024) CSCO guidelines for colorectal cancer version 2024: updates and discussions. Chin J Cancer Res 36:233–239 38988483 10.21147/j.issn.1000-9604.2024.03.01 PMC11230882

[5] Lee H, Yoo SY, Park IJ et al (2022) The prognostic reliability of lymphovascular invasion for patients with T3N0 colorectal cancer in adjuvant chemotherapy decision making. Cancers (Basel) 14:2833 35740498 10.3390/cancers14122833 PMC9221415

[6] Knijn N, van Exsel UEM, de Noo ME, Nagtegaal ID (2018) The value of intramural vascular invasion in colorectal cancer—a systematic review and meta-analysis. Histopathology 72:721–728 28960400 10.1111/his.13404

[7] Huh JW, Lee JH, Kim HR, Kim YJ (2013) Prognostic significance of lymphovascular or perineural invasion in patients with locally advanced colorectal cancer. Am J Surg 206:758–763 23835209 10.1016/j.amjsurg.2013.02.010

[8] Amin MB, Greene FL, Edge SB et al (2017) The eighth edition AJCC cancer staging manual: continuing to build a bridge from a population-based to a more “personalized” approach to cancer staging. CA Cancer J Clin 67:93–99 28094848 10.3322/caac.21388

[9] Horvat N, Carlos Tavares Rocha C, Clemente Oliveira B, Petkovska I, Gollub MJ (2019) MRI of rectal cancer: tumor staging, imaging techniques, and management. Radiographics 39:367–387 30768361 10.1148/rg.2019180114 PMC6438362

[10] Gao Y, Li J, Ma X et al (2019) The value of four imaging modalities in diagnosing lymph node involvement in rectal cancer: an overview and adjusted indirect comparison. Clin Exp Med 19:225–234 30900099 10.1007/s10238-019-00552-z

[11] Gillies RJ, Kinahan PE, Hricak H (2016) Radiomics: images are more than pictures, they are data. Radiology 278:563–577 26579733 10.1148/radiol.2015151169 PMC4734157

[12] Abbaspour E, Karimzadhagh S, Monsef A, Joukar F, Mansour-Ghanaei F, Hassanipour S (2024) Application of radiomics for preoperative prediction of lymph node metastasis in colorectal cancer: a systematic review and meta-analysis. Int J Surg 110:3795–3813 38935817 10.1097/JS9.0000000000001239 PMC11175807

[13] Xu F, Hong J, Wu X (2025) An integrative clinical and intra- and peritumoral MRI radiomics nomogram for the preoperative prediction of lymphovascular invasion in rectal cancer. Acad Radiol 32:3989–4001 40044546 10.1016/j.acra.2025.02.019

[14] O’Connor JP, Rose CJ, Waterton JC, Carano RA, Parker GJ, Jackson A (2015) Imaging intratumor heterogeneity: role in therapy response, resistance, and clinical outcome. Clin Cancer Res 21:249–257 25421725 10.1158/1078-0432.CCR-14-0990 PMC4688961

[15] Shang Y, Zeng Y, Luo S et al (2024) Habitat imaging with tumoral and peritumoral radiomics for prediction of lung adenocarcinoma invasiveness on preoperative chest CT: a multicenter study. AJR Am J Roentgenol 223:e2431675 39140631 10.2214/AJR.24.31675

[16] Wang Y, Xie B, Wang K et al (2025) Multi-parametric MRI habitat radiomics based on interpretable machine learning for preoperative assessment of microsatellite instability in rectal cancer. Acad Radiol 32:3975–3988 40016002 10.1016/j.acra.2025.02.009

[17] Shi S, Jiang T, Liu H et al (2025) Habitat radiomics based on MRI for predicting metachronous liver metastasis in locally advanced rectal cancer: a two‑center study. Acad Radiol 32:3370–3383 40204586 10.1016/j.acra.2025.02.046

[18] Su Y, Zhao H, Lyu Z et al (2025) Quantification of intratumoral heterogeneity based on habitat analysis for preoperative assessment of lymphovascular invasion in colorectal cancer. Acad Radiol 32:4532–4543 10.1016/j.acra.2025.03.014 40175205

[19] Xie P, Huang Q, Zheng L et al (2025) Sub-region based histogram analysis of amide proton transfer-weighted MRI for predicting tumor budding grade in rectal adenocarcinoma: a prospective study. Eur Radiol 35:1382–1393 39500798 10.1007/s00330-024-11172-x

[20] Zhou M, Chen M, Luo M, Chen M, Huang H (2025) Pathological prognostic factors of rectal cancer based on diffusion-weighted imaging, intravoxel incoherent motion, and diffusion kurtosis imaging. Eur Radiol 35:979–988 39143248 10.1007/s00330-024-11025-7

[21] Jensen JH, Helpern JA, Ramani A, Lu H, Kaczynski K (2005) Diffusional kurtosis imaging: the quantification of non-Gaussian water diffusion by means of magnetic resonance imaging. Magn Reson Med 53:1432–1440 15906300 10.1002/mrm.20508

[22] Granata V, Fusco R, Reginelli A et al (2019) Diffusion kurtosis imaging in patients with locally advanced rectal cancer: current status and future perspectives. J Int Med Res 47:2351–2360 31032670 10.1177/0300060519827168 PMC6567719

[23] Hu S, Peng Y, Wang Q et al (2022) T2*-weighted imaging and diffusion kurtosis imaging (DKI) of rectal cancer: correlation with clinical histopathologic prognostic factors. Abdom Radiol (NY) 47:517–529 34958406 10.1007/s00261-021-03369-1

[24] Cui Y, Yang X, Du X, Zhuo Z, Xin L, Cheng X (2018) Whole-tumour diffusion kurtosis MR imaging histogram analysis of rectal adenocarcinoma: correlation with clinical pathologic prognostic factors. Eur Radiol 28:1485–1494 29063250 10.1007/s00330-017-5094-3

[25] Ma YR, Zhang ZW, Wen ZQ et al (2025) Application of diffusion kurtosis imaging in differentiating T0-T1 from T2 rectal tumors. Cancer Imaging 25:124 41185075 10.1186/s40644-025-00947-0 PMC12581579

[26] Mc Entee PD, Shokuhi P, Rogers AC et al (2022) Extramural venous invasion (EMVI) in colorectal cancer is associated with increased cancer recurrence and cancer-related death. Eur J Surg Oncol 48:1638–1642 35249791 10.1016/j.ejso.2022.02.013

[27] Luyang H, Zeng F, Lei Y, He Q, Zhou Y, Xu J (2025) Bidirectional role of neutrophils in tumor development. Mol Cancer 24:22 39819428 10.1186/s12943-025-02228-7 PMC11737241

[28] Chiang JM, Chang CJ, Jiang SF et al (2017) Pre-operative serum albumin level substantially predicts post-operative morbidity and mortality among patients with colorectal cancer who undergo elective colectomy. Eur J Cancer Care. 10.1111/ecc.12403 10.1111/ecc.12403 26526411

[29] Kratz F (2008) Albumin as a drug carrier: design of prodrugs, drug conjugates and nanoparticles. J Control Release 132:171–183 18582981 10.1016/j.jconrel.2008.05.010

[30] Vural S, Muhtaroğlu A, Uygur FA (2023) The relationship between preoperative CEA and CA19-9 status and patient characteristics and lymph node involvement in early-stage colon cancer. Eur Rev Med Pharmacol Sci 27:4563–4569 37259737 10.26355/eurrev_202305_32462

[31] Kildusiene I, Dulskas A, Smailyte G (2024) Value of combined serum CEA, CA72-4, and CA19-9 marker detection in diagnosis of colorectal cancer. Tech Coloproctol 28:33 38358422 10.1007/s10151-023-02873-4

[32] Paragomi P, Zhang Z, Abe SK et al (2024) Body mass index and risk of colorectal cancer incidence and mortality in Asia. JAMA Netw Open 7:e2429494 39196559 10.1001/jamanetworkopen.2024.29494 PMC11358861

[33] Jardim-Perassi BV, Huang S, Dominguez-Viqueira W et al (2019) Multiparametric MRI and coregistered histology identify tumor habitats in breast cancer mouse models. Cancer Res 79:3952–3964 31186232 10.1158/0008-5472.CAN-19-0213 PMC6677627

