# Supplementary material for: An interpretable machine learning model combining MRI-DKI habitat radiomic features and clinical biomarkers for noninvasive prediction of lymphatic metastasis in rectal cancer: a prospective study
Source: Insights Imaging. 2026 Mar 25;17:79. doi: 10.1186/s13244-026-02243-2 (PMC13018487; doi:10.1186/s13244-026-02243-2)
Supplement: Supplementary file 1 — Supplementary information [file 13244_2026_2243_MOESM1_ESM.pdf]

An Interpretable Machine Learning Model Combining MRI-DKI Habitat Radiomic Features and Clinical Biomarkers for Noninvasive Prediction of Lymphatic Metastasis in Rectal Cancer: A Prospective Study

ELECTRONIC SUPPLEMENTARY MATERIAL

Appendix E1

Clinical baseline data of patients

Baseline clinical data were prospectively collected from the patient's electronic medical records, including age, sex, BMI, History of colorectal polyps, serum lipid metabolism levels, complete blood count, carcinoembryonic antigen (CEA), carbohydrate antigen 19-9 (CA19-9), carbohydrate antigen 7-24 (CA7-24), MRI-based TNM staging, mesorectal fascia (MRF) involvement, extramural venous invasion (EMVI) status, and clinical staging. Based on the normal ranges used at our institution, the threshold levels for CEA, CA19-9, and CA7-24 were set at 5 ng/mL, 37.0 U/mL, and 6.9 U/mL, respectively. TNM staging was reclassified according to the 7th edition of the American Joint Committee on Cancer (AJCC)/International Union Against Cancer (UICC) Cancer Staging Manual. Tumor Location: Based on the distance from the lower edge of the tumor to the anal verge, tumors were classified as follows: ≤5 cm as low RC, 5-10 cm as middle RC, and ≥10 cm as high RC. Early-onset rectal cancer (EORC) is defined as rectal cancer patients younger than 50 years old, and late-onset rectal cancer (LORC) is defined as rectal cancer patients aged 50 years or older [1, 2]. The clinical baseline data of the patients included in this study were not missing. The detailed classification of the above clinical and immune-inflammatory biomarkers data is shown in Table 1.

Appendix E2

Immune-inflammatory biomarkers calculation

Preoperative complete blood count results from patients were collected and analyzed to calculate the corresponding immune-inflammatory biomarkers. The specific formulas are as follows: Systemic Inflammation Response Index (SIRI) = Neutrophil count (N) * Monocyte count (M) / Lymphocyte count (L), NLR= N/L, PLR= Platelet count (PLT) /L, MLR= M/L, SII= PLT * N/L, ENLR= Eosinophil count (EOS)/L, HALP= Hemoglobin (HB) * Albumin (ALB) * L/PLT, PNI= ALB+5 * L, Aggregate Inflammation Systemic Index (AISI)= N * PLT /L, NHR=N/high density lipoprotein (HDL), MHR=M/HDL, LHR=L/HDL, PLR=PLT/HDL.

Appendix E3

LVI and LNM pathological evaluation criteria

Two pathologists (W.C. and L.W.), with 10 and 8 years of experience in diagnosing RC, respectively, and blinded to the patients' imaging information, evaluated the postoperative RC and lymph node resection specimens. Any discrepancies were resolved through consensus. LNM positivity was defined as the presence of cancer cells within lymph node tissue. LVI positivity was defined as the identification of tumor cells within lymphatic or blood vessels, thrombus formation within the vascular endothelium, or tumor cell infiltration into the lymphatic vessel wall, as determined by pathological analysis following radical RC surgery [3].

Appendix E4

Protocols and procedures

MRI examinations were performed using 3.0T MRI scanners (Skyra, Siemens, Germany; Elition, Philips, the Netherlands), equipped with phased-array coils. Standardized protocols were followed to acquire oblique, sagittal, and coronal views. T2WI images were used as the baseline reference, and DKI parametric maps were applied for further analysis. For the Siemens Skyra 3.0T scanner, the oblique axial DKI parameters were as follows: repetition time (TR)/echo time (TE), 2009 ms/102 ms; field of view (FOV), 280 mm×220 mm; slice thickness, 6 mm; slice gap, 3 mm. For the Philips Elition 3.0T scanner, the oblique axial DKI parameters were: TR/TE, 4900 ms/93 ms; FOV, 304 mm×380 mm; slice thickness, 4 mm; slice gap, 2.4 mm. The b-values were set at 0, 1000, and 2000 s/mm².

Appendix E5

Image preprocessing and segmentation

First, N4 bias field correction was applied to the DKI parameter maps to remove low-frequency intensity inhomogeneity artifacts. Second, intensity normalization was performed using z-score standardization, with the mean set to 0 and the standard deviation set to 1, to minimize inter-scan intensity variations. Third, B-spline interpolation was used to resample all images to a uniform voxel size of 1×1×3 $mm^3$to ensure spatial consistency across scans. Finally, absolute gray-level discretization with a fixed bin width of 25 was applied to standardize gray-level distribution and ensure consistent radiomics feature extraction.Two radiologists with 6 (Reader 1, X.M.M) and 7 (Reader 2, Y.M.J) years of experience in abdominal diagnosis independently evaluated the MRI imaging features. They were blinded to all clinical and histopathological information of the patients; any discrepancies were resolved through consensus. Two radiologists manually delineated the tumor VOI on a slice-by-slice basis using ITK-SNAP software (version 3.8; http://www.itksnap.org), with T2WI as the anatomical reference and segmentation performed on the MD maps. The VOIs were then mapped onto the MK maps. After an interval of one month, two radiologists randomly selected 20 patients to repeat tumor VOI segmentation and extract conventional radiomic features. The consistency of VOI segmentation and the robustness of the features were evaluated using Dice coefficients and the intraclass correlation of conventional radiomic features.

Appendix E6

Feature extraction and selection

First, conventional radiomic features with good consistency were screened, and those with inter-observer ICC values greater than 0.75 were retained. Second, analysis of variance (ANOVA), Mann-Whitney U-test (with Bonferroni correction), and minimum redundancy maximum relevance (mRMR) were performed for the preliminary selection of conventional and habitat radiomic features. Ultimately, the top 200 radiomic features significantly associated with lymphatic metastasis risk, with all *P*-values less than 0.05, were retained for subsequent analysis. Third, mutual information analysis was conducted to further identify features strongly correlated with the outcome. Fourth, Pearson correlation analysis was used to eliminate redundant features with an average absolute correlation coefficient greater than 0.9. Finally, the least absolute shrinkage and selection operator (LASSO) algorithm, combined with five-fold cross-validation, was applied to select features with nonzero coefficients. To further reduce overfitting and assess the robustness of the selected features, permutation testing was performed on the final feature set to ensure model stability and minimize the risk of overfitting.

Appendix E7

Construction of clinical and combined models

In this study, logistic regression analyses were conducted to identify independent predictive factors for assessing LVI and LNM, respectively. Variables with a *P*-value less than 0.1 in the univariate logistic regression analysis were included in the multivariate logistic regression. Variables with a *P*-value less than 0.05 in the multivariate analysis were considered optimal predictors and were used to construct the clinical model. A combined model (Model 3) was developed by integrating clinical risk factors, the conventional radiomics model, and the habitat-based model. Additionally, we compared the predictive performance of clinical benchmark models, including MRI-reported T and N stages as well as the combined CEA and CA19-9. The detailed results are provided in Supplementary material 2_Fig.S7.

Appendix E8

SHAP interpretability analysis of Model3

We calculated the overall and individual Shapley values for the Model 3 interpretation and clinical application. In the overall visualization, the SHAP beeswarm plot (Fig. 5b and 5g) shows each feature's positive or negative effects on the prediction probability in red and blue. The positive SHAP values indicate that a feature pushes the model's prediction toward a higher risk of LVI or LNM, whereas negative SHAP values indicate that the feature shifts the prediction toward a lower risk of lymphatic metastasis. The SHAP bar chart (Fig. 5a and 5f) shows the weights of the most important characteristics. In predicting LVI, the features with the highest weights were MK_Habitat_Score and EMVI, while in predicting LNM, PNI and EMVI had the greatest weights. The SHAP heatmap plot (Fig. 5d and 5e) shows each feature's direction and intensity of influence in all model cases, whereas the SHAP decision plot (Fig. 5c and 5h) shows the impact process of each significant feature on the final predicted probability. For LVI prediction, wavelet_LLH_glcm_Idn_h1 and EMVI were the features contributing most positively to lymphatic metastasis risk, while NHR contributed most negatively. For LNM prediction, EMVI and CA19-9 were the features with the strongest positive contribution to lymphatic metastasis risk, whereas PNI had the strongest negative contribution. The SHAP effort plot (Fig.6) shows each feature's positive and negative effects on predictive outcomes in a single case. The base value represents the basic prediction probability of the model, and f (x) represents its final prediction probability.

Appendix E9

Analysis of the SHAP results at the individual level in Fig. 6

Patient 1：

LVI+: MK_Habitat_score=0.8, SHAP value=+0.5; MD_Habitat_score=0.75, SHAP value=+0.28; T_score=0.58, SHAP value=-0.04; EMVI=Positive, SHAP value=+0.57; NHR=8.07, SHAP value=-0.45; f(x)=1.45; E[f(x)]=0.593.

LNM+：MK_Habitat_score_n=0.64, SHAP value=+0.48; MD_Habitat_score_n=0.46, SHAP value=+0.02; T_score_n=0.49, SHAP value=+0.05; CA19-9=1 (≥37 U/mL), SHAP value=+1.27; BMI=1 (22.86 Kg/m^2), SHAP value=-0.2; EMVI=Positive, SHAP value=+0.53; PNI=8.07, SHAP value=-0.17; f(x)=1.76, E[f(x)]=-0.211.

Patient 2：

LVI-: MK_Habitat_score=0.27, SHAP value=-0.97; MD_Habitat_score=0.36, SHAP value=-0.44; T_score=0.53, SHAP value=-0.12; EMVI=Negative, SHAP value=-0.35; NHR=4.92, SHAP value=+0.01; f(x)=-1.27; E[f(x)]=0.593.

LNM-：MK_Habitat_score_n=0.25, SHAP value=-0.39; MD_Habitat_score_n=0.38, SHAP value=-0.03; T_score_n=0.33, SHAP value=-0.10; CA19-9=0 (＜37 U/mL), SHAP value=-0.19; BMI=1 (20.07 Kg/m^2), SHAP value=-0.2; EMVI=Negative, SHAP value=-0.33; PNI=39.66, SHAP value=+0.65; f(x)=-0.80, E[f(x)]=-0.211.

Patient 3：

LVI+: MK_Habitat_score=0.88, SHAP value=+0.74; MD_Habitat_score=0.77, SHAP value=+0.31; T_score=0.638, SHAP value=+0.03; EMVI=Positive, SHAP value=+0.57; NHR=11.93, SHAP value=-1.01; f(x)=1.23; E[f(x)]=0.593.

LNM-：MK_Habitat_score_n=0.70, SHAP value=+0.61; MD_Habitat_score_n=0.42, SHAP value=-0.01; T_score_n=0.47, SHAP value=+0.04; CA19-9=0 (＜37 U/mL), SHAP value=-0.19; BMI=1 (22.15 Kg/m^2), SHAP value=-0.2; EMVI=Positive, SHAP value=+0.53; PNI=47.78, SHAP value=-0.17; f(x)=0.39, E[f(x)]=-0.211. Since f(x) > E[f(x)], the model incorrectly predicted the patient as LNM+, but the patient is actually LNM-.

Patient 4：

LVI-: MK_Habitat_score=0.45, SHAP value=-0.46; MD_Habitat_score=0.26, SHAP value=-0.62; T_score=0.456, SHAP value=-0.21; EMVI=Negative, SHAP value=-0.35; NHR=9.37, SHAP value=-0.64; f(x)=-1.67; E[f(x)]=0.593.

LNM+：MK_Habitat_score_n=0.41, SHAP value=-0.04; MD_Habitat_score_n=0.42, SHAP value=-0.01; T_score_n=0.39, SHAP value=-0.05; CA19-9=1 (≥37 U/mL), SHAP value=+1.27; BMI=1 (23.88 Kg/m^2), SHAP value=-0.2; EMVI=Negative, SHAP value=-0.33; PNI=33.65, SHAP value=+1.27; f(x)=1.70, E[f(x)]=-0.211.

E[f(x)] represents the benchmark function value (the average model output value of all samples). f(x)＞E[f(x)]，The possibility of predicting LVI+ or LNM+ is greater.

References

1. Sinicrope FA (2022) Increasing Incidence of Early-Onset Colorectal Cancer. N Engl J Med 386:1547-1558.
2. Zaborowski AM, Abdile A, Adamina M et al (2021) Characteristics of Early-Onset vs Late-Onset Colorectal Cancer: A Review. JAMA Surg 156:865-874.
3. Tong P, Sun D, Chen G, Ni J, Li Y (2023) Biparametric magnetic resonance imaging-based radiomics features for prediction of lymphovascular invasion in rectal cancer. BMC Cancer 23:61.

Supplementary material 2

An Interpretable Machine Learning Model Combining MRI-DKI Habitat Radiomic Features and Clinical Biomarkers for Noninvasive Prediction of Lymphatic Metastasis in Rectal Cancer: A Prospective Study

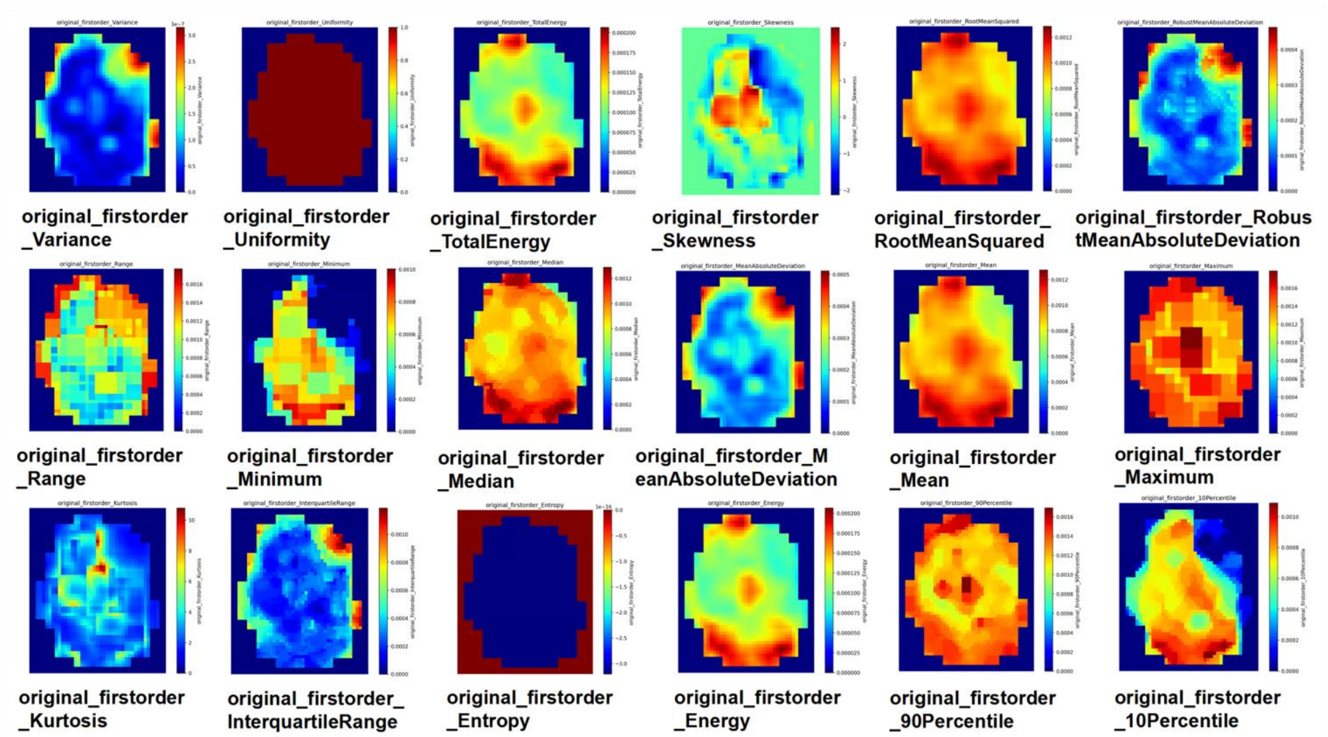

a

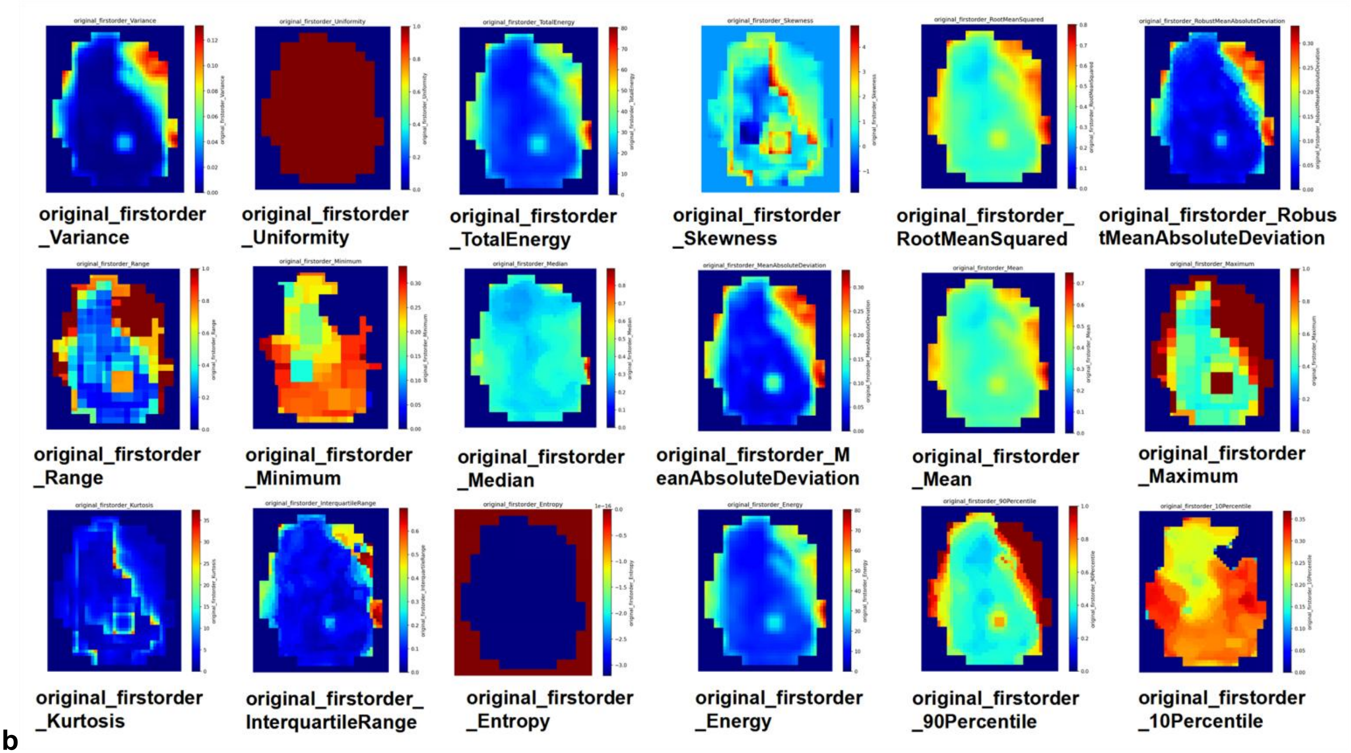

b

Fig.S1 18 first-order histogram radiomics features. (a) 18 first-order histogram radiomics features of the MD plot; (b) 18 first-order histogram radiomics features of the MK plot.

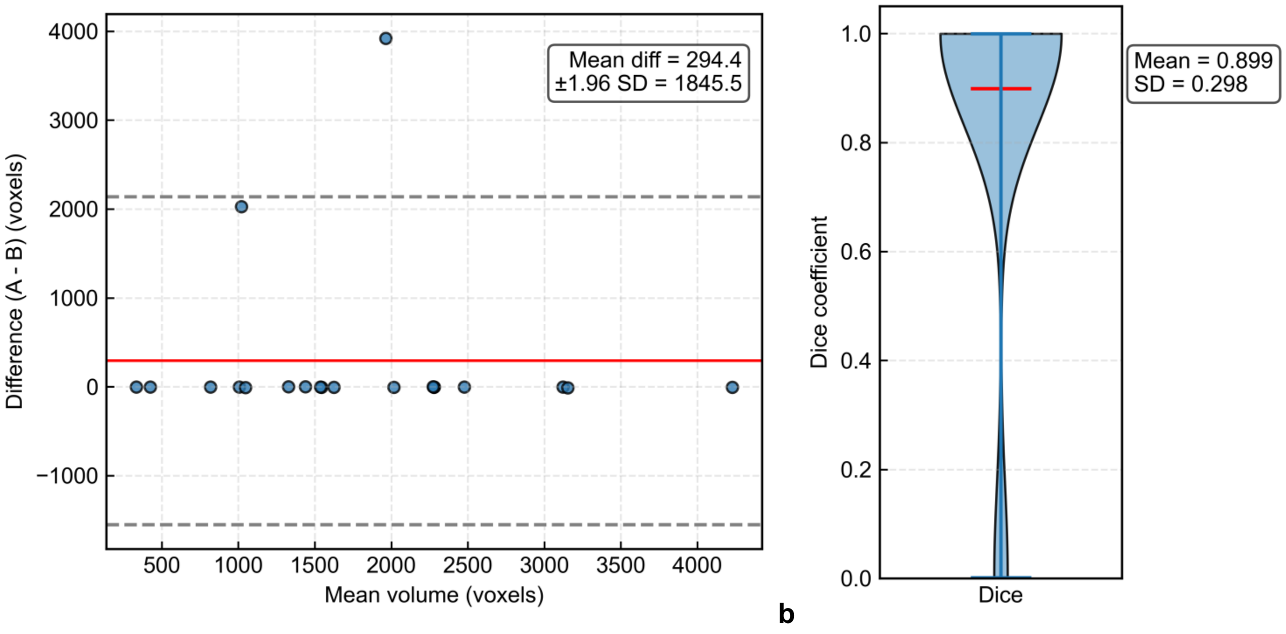

Fig.S2 Consistency and overlap analysis of segmentation results. (a) Bland-Altman plot illustrating the agreement of tumor volume measurements between two observers. The solid line indicates the mean difference, and the dashed lines represent the 95% limits of agreement. (b) Violin plot showing the distribution of Dice similarity coefficients between the two observers. The box indicates the median and interquartile range, while the shape reflects the data density. The mean Dice coefficient was 0.899, with a standard deviation of 0.298.

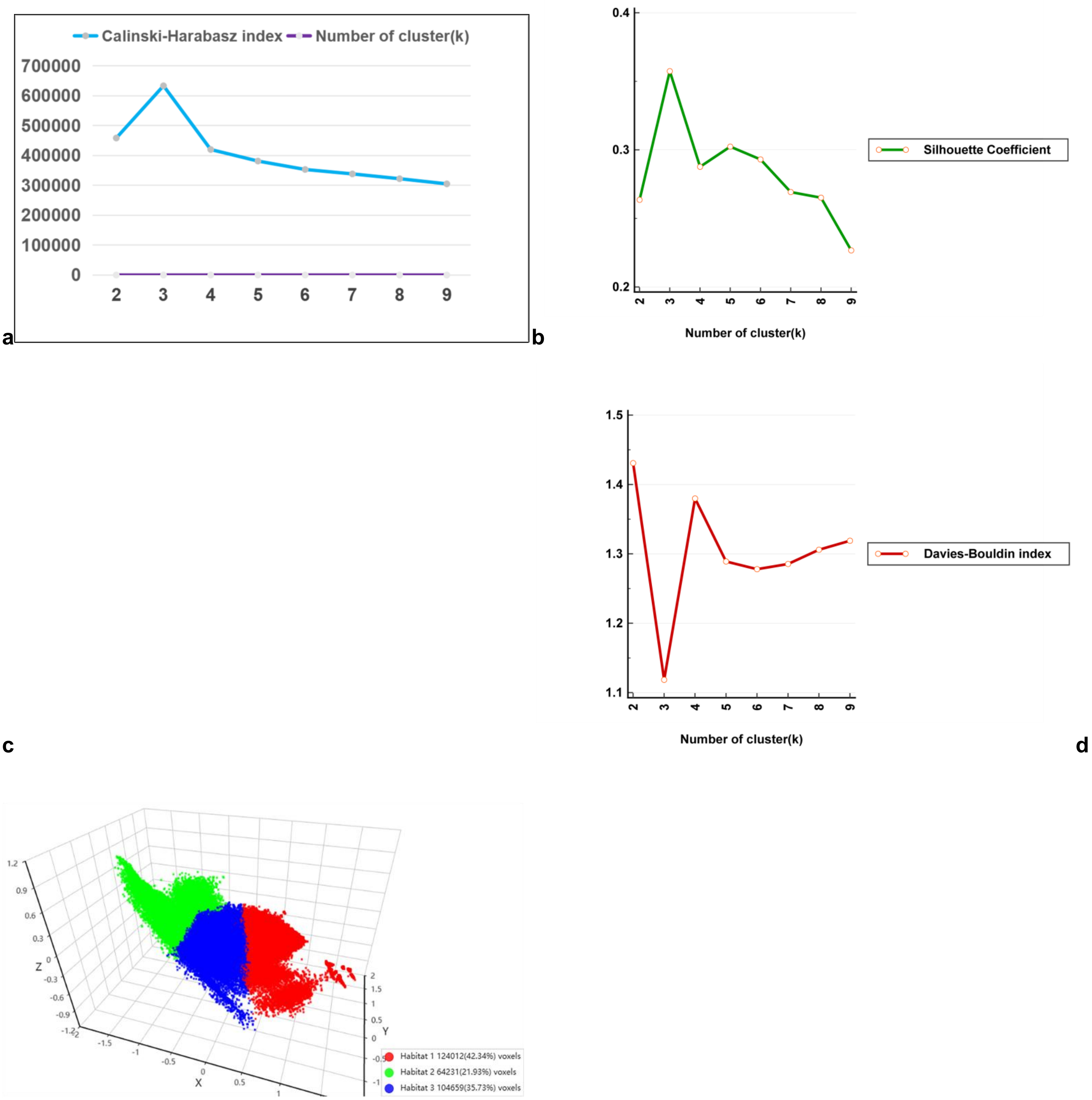

Fig.S3 Methods and results of habitat clustering and segmentation. (a) The Calinski–Harabasz index, (b) the Silhouette Coefficient, and (c) the Davies-Bouldin index confirmed that the optimal number of clustered subregions was three; (d) The three-dimensional subregion segmentation images show the voxel counts and corresponding proportions for each subregion.

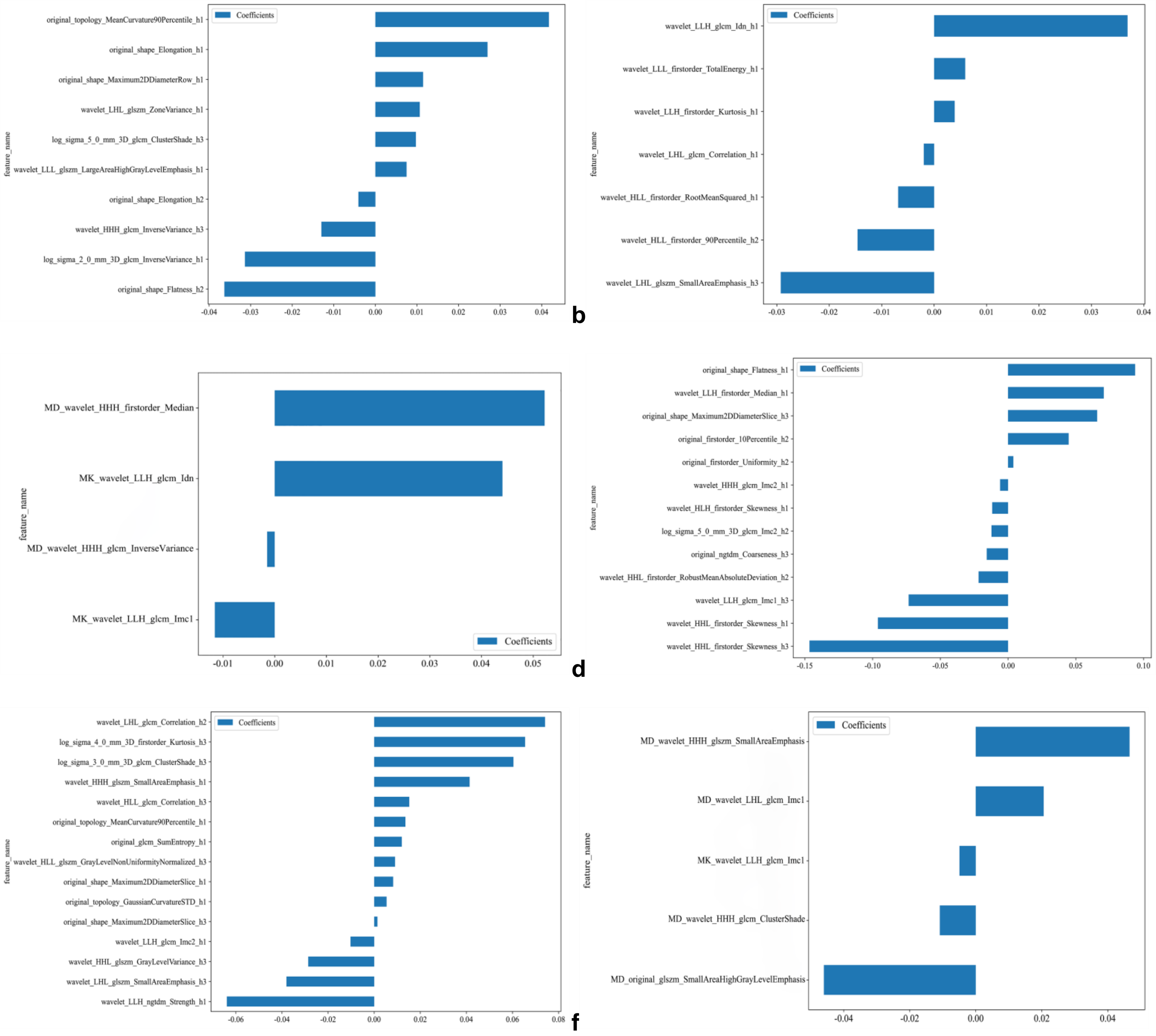

Fig.S4 Optimal radiomics feature weights for different models. (a–c) show the feature weight coefficients of the MD map habitat model, MK map habitat model, and MD+MK map conventional radiomics model for predicting LVI, respectively; (d–f) show the feature weight coefficients of the MD map habitat model, MK map habitat model, and MD+MK map conventional radiomics model for predicting LNM, respectively.

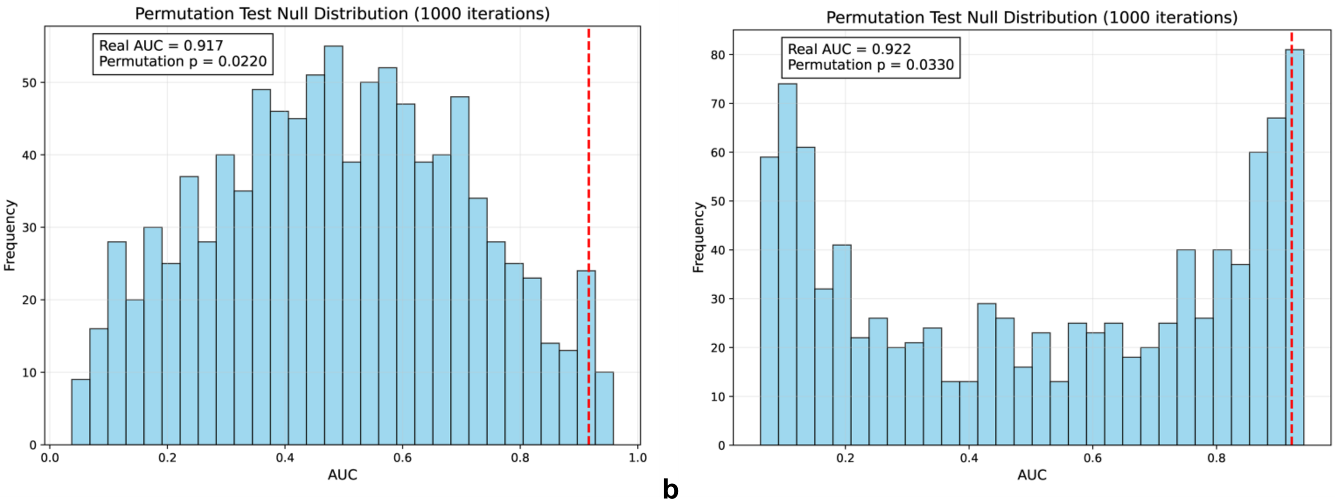

Fig.S5 Permutation test analysis. (a) For predicting LNM, the histogram illustrates the null distribution of AUC values obtained from 1,000 permutation iterations. The red line denotes the actual model AUC (0.922), with a permutation *P*-value of 0.033. (b) For predicting LVI, the histogram shows the null distribution of AUC values generated from 1,000 permutations, with the actual model AUC (0.917) marked in red (permutation *P*=0.022). These results demonstrate that the model exhibits significant predictive performance and robustness, with a low risk of overfitting.

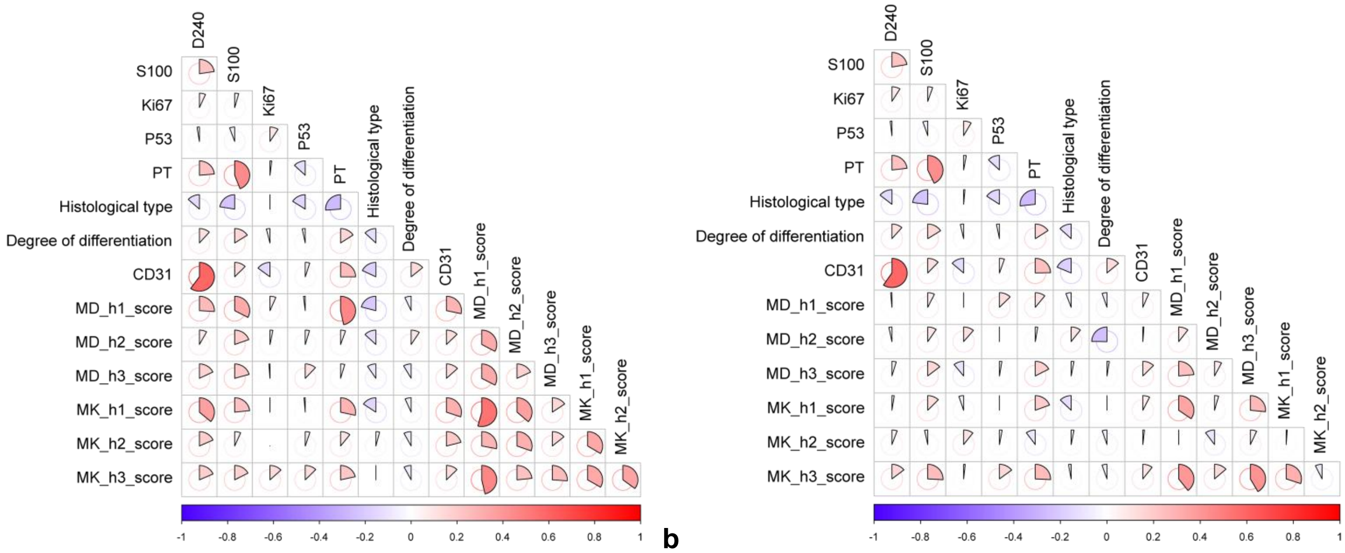

Fig. S6 Correlation analysis. (a) and (b) are the heat maps for analyzing the correlation between Radiomics signatures and pathological features in different subregions in predicting LVI and LNM, respectively.

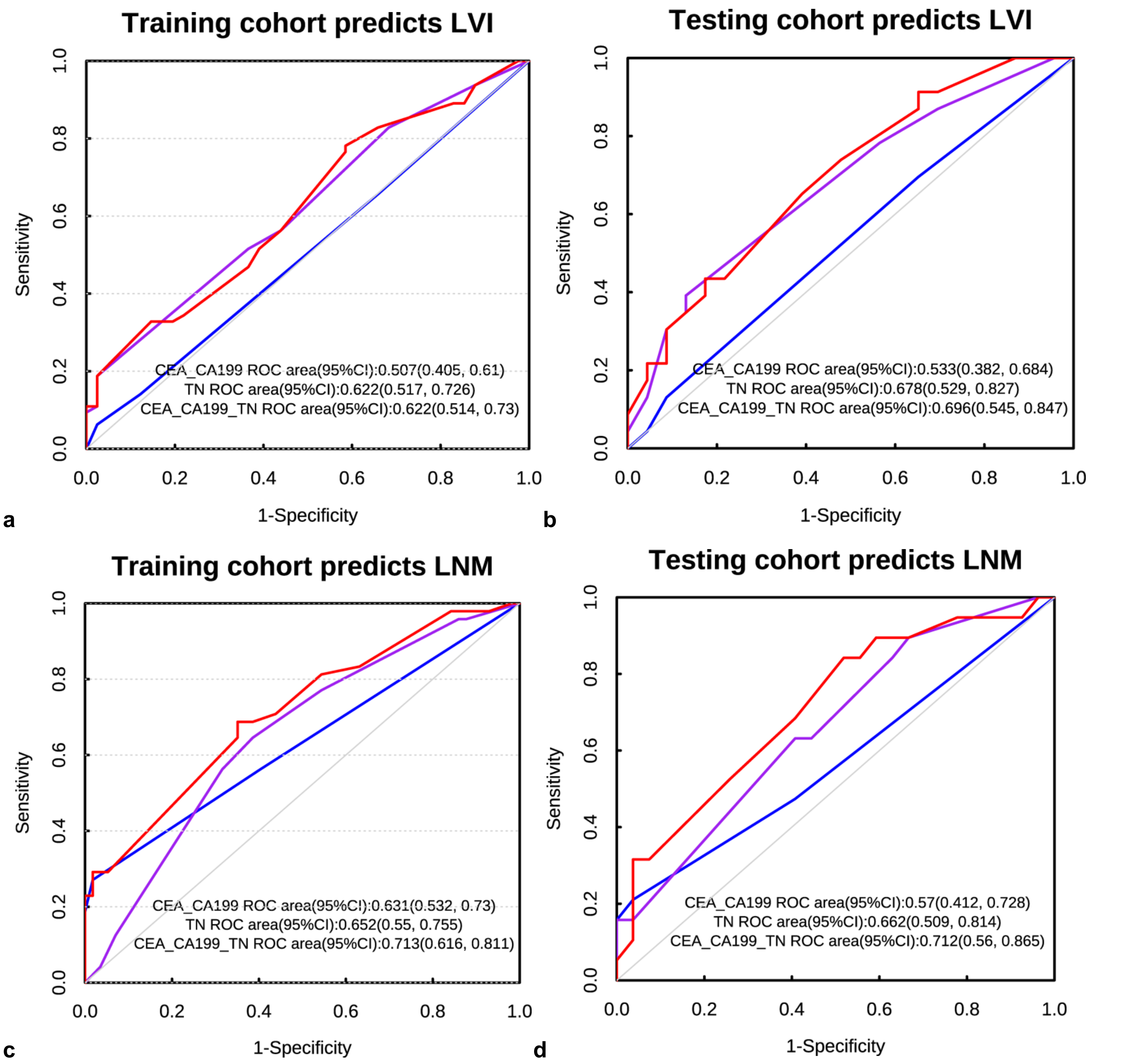

Fig. S7 Comparison of ROC performance of clinical benchmark models. (a) and (b) predict rectal cancer LVI for the training and the testing cohorts, respectively; (c) and (d) predict rectal cancer LNM for the training and the testing cohorts, respectively.

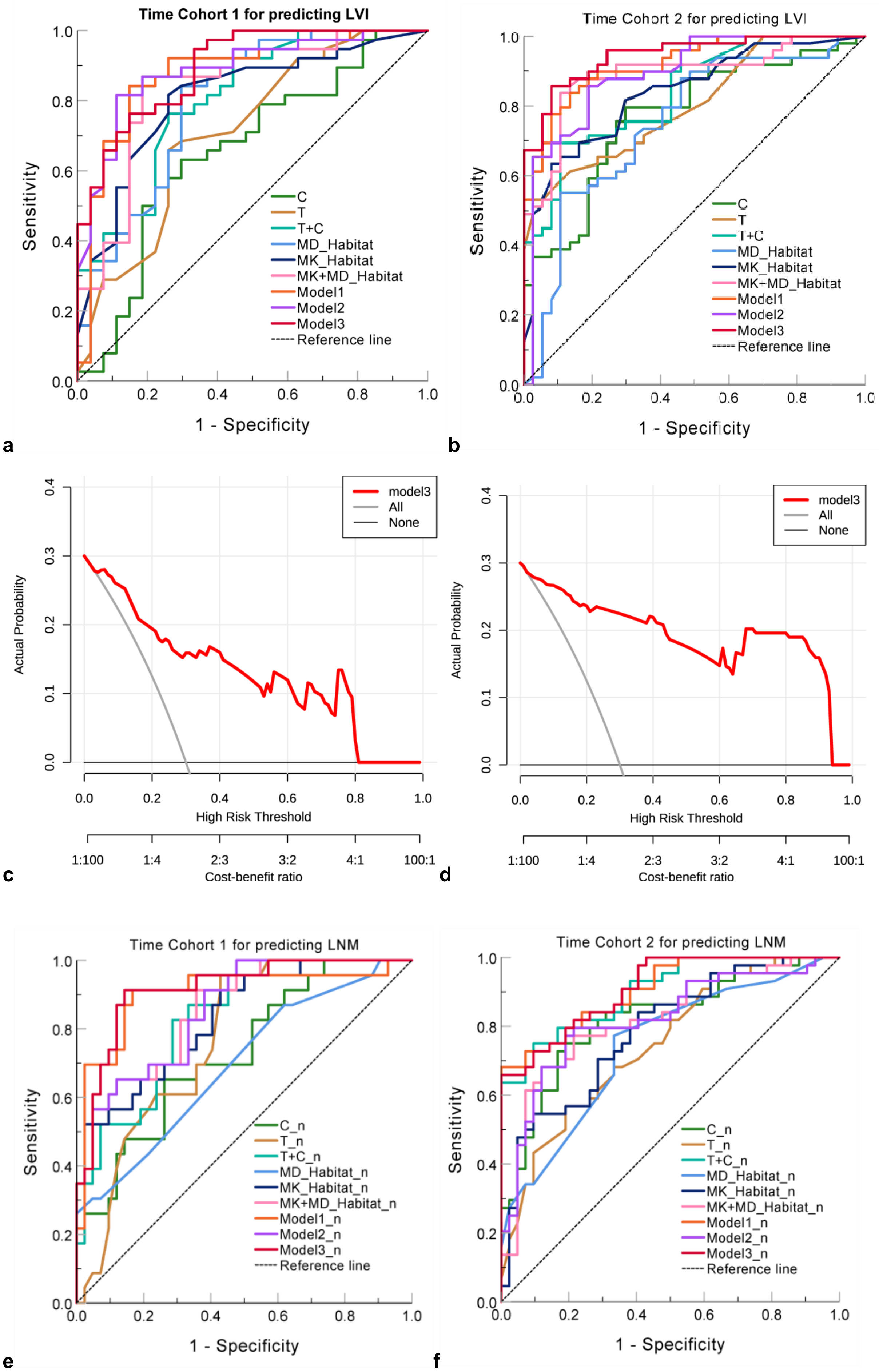

Insights Imaging (2026) Peng L, Li F, Zhang F, et al.

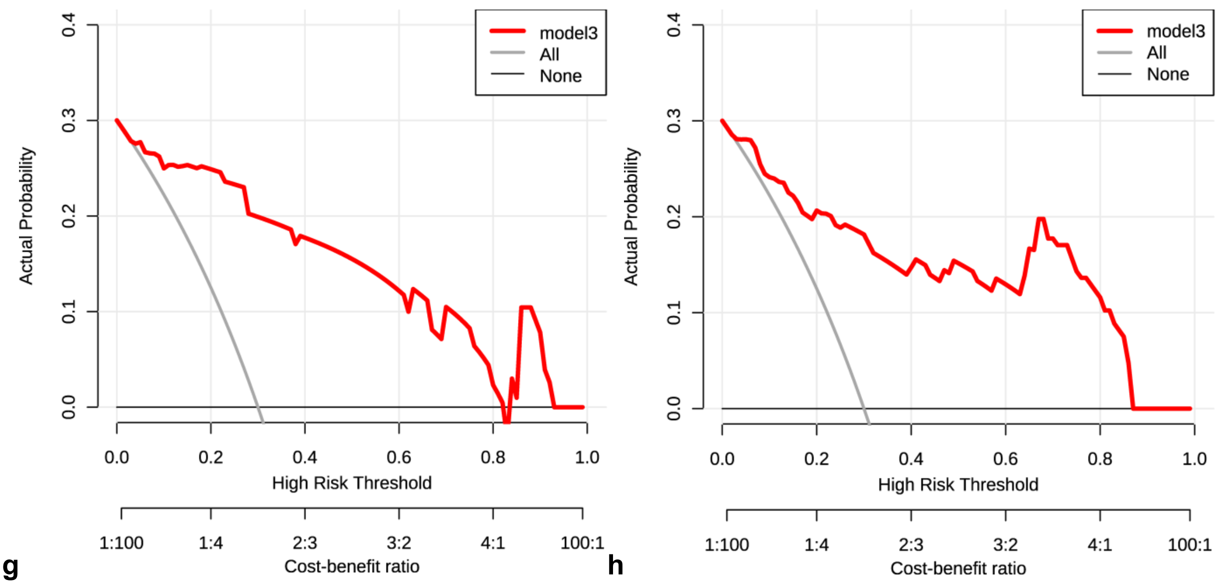

Fig.S8 Comparison of predictive performance across the two time cohorts and decision curve analysis (DCA) for Model 3. (a–d) correspond to LVI prediction: (a) and (c) show the ROC curve and DCA for Model 3 in Time Cohort 1, while (b) and (d) show the corresponding results in Time Cohort 2. (e–h) correspond to LNM prediction: (e) and (f) present the ROC curve and DCA for Model 3 in Time Cohort 1, while (g) and (h) display the results in Time Cohort 2.

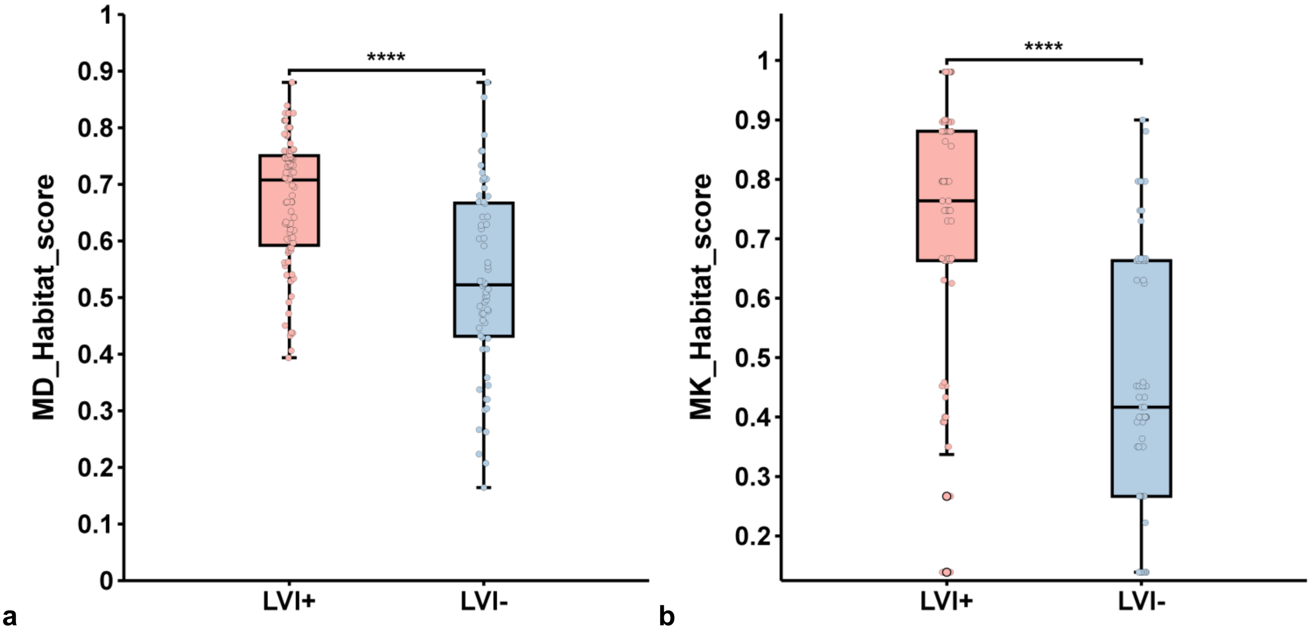

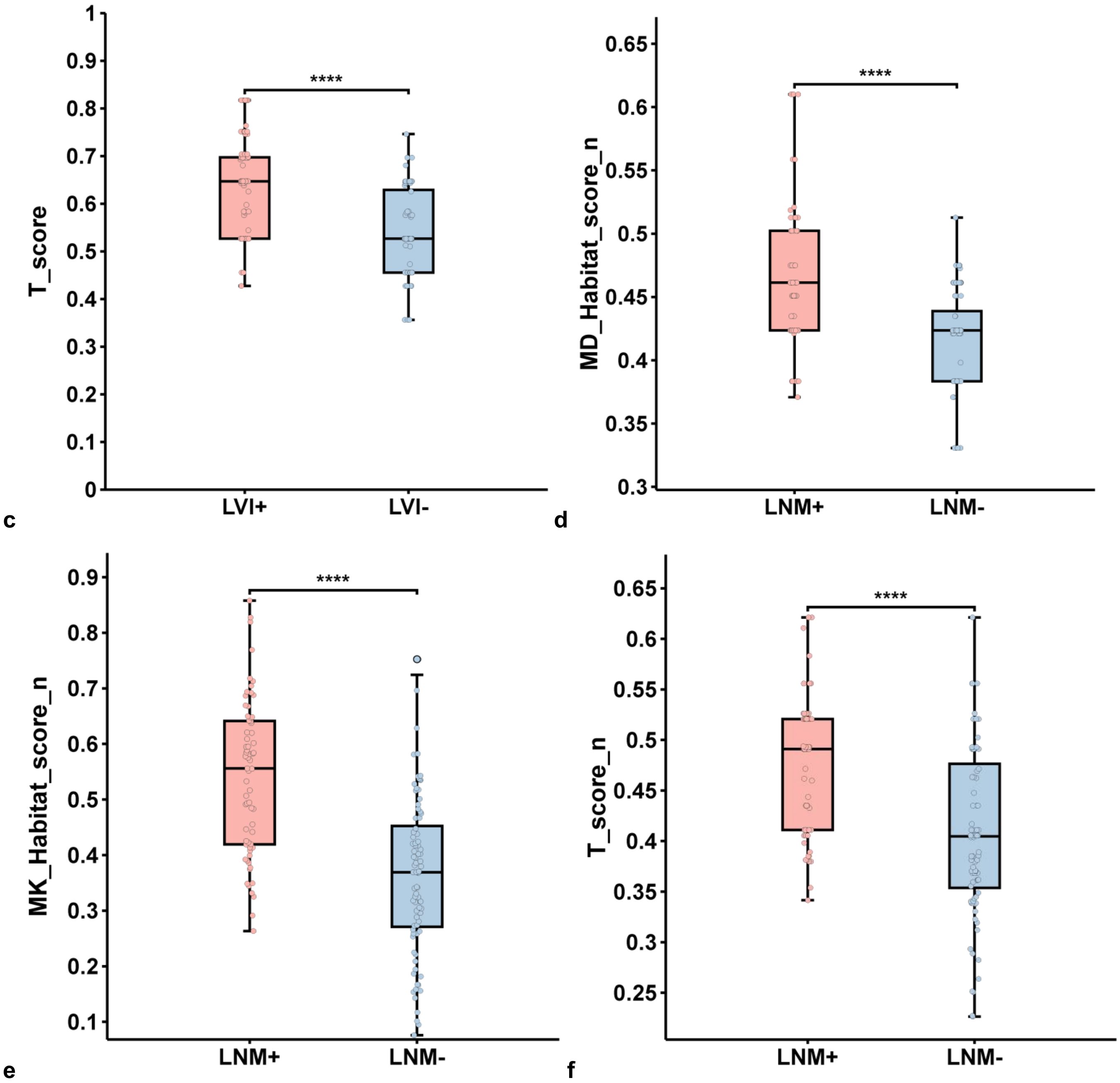

Fig.S9 Comparison of radiomics scores. (a–c) show that MD_Habitat_Score, MK_Habitat_Score, and T_Score are significantly higher in the LVI+ group than in the LVI- group (*P* < 0.001); (d–f) show that MD_Habitat_Score, MK_Habitat_Score, and T_Score are significantly higher in the LNM+ group than in the LNM- group (*P* < 0.001). **** indicates *P* < 0.001.

**Table S1** Evaluation of inter-class consistency in optimal conventional radiomic features.		
**Features**	**ICC**	**95%CI**
MD_wavelet_HHH_firstorder_Median	0.996	0.989-0.998
MD_wavelet_HHH_glcm_InverseVariance	0.997	0.994-0.999
MK_wavelet_LLH_glcm_Idn	0.996	0.990-0.999
MK_wavelet_LLH_glcm_Imc1	0.999	0.998-1.000
MD_wavelet_HHH_glszm_SmallAreaEmphasis	0.991	0.977-0.996
MD_wavelet_LHL_glcm_Imc1	0.998	0.995-0.999
MD_original_glszm_SmallAreaHighGrayLevelEmphasis	0.999	0.996-1.000
MD_wavelet_HHH_glcm_ClusterShade	0.994	0.986-0.998
MK_wavelet_LLH_glcm_Imc1	0.999	0.998-1.000
***Note--**95%CI, 95% confidence interval; ICC, intraclass correlation coefficient		

Table S2 Comparison of ROC performance of different models at different time periods.									
**Research on predicting LVI in Rectal cancer**									
**Cohort**	**Model**	**AUC**	**95%CI**	**Accuracy**	**Sensitivity**	**Specificity**	**PPV**	**NPV**	**Threshold**
Time Cohort 1	C	0.659	0.552-0.796	0.662	0.632	0.704	0.750	0.576	0.612
Time Cohort 1	MD_Habitat	0.801	0.689-0.913	0.785	0.842	0.704	0.800	0.760	0.529
Time Cohort 1	MK_Habitat	0.810	0.702-0.918	0.785	0.816	0.741	0.816	0.741	0.561
Time Cohort 1	MK+MD_Habitat	0.841	0.737-0.945	0.831	0.842	0.815	0.865	0.786	0.537
Time Cohort 1	T	0.713	0.580-0.846	0.692	0.658	0.741	0.781	0.606	0.582
Time Cohort 1	T+C	0.808	0.702-0.914	0.754	0.763	0.741	0.806	0.690	0.584
Time Cohort 1	Model1	0.877	0.789-0.965	0.846	0.842	0.852	0.889	0.793	0.536
Time Cohort 1	Model2	0.887	0.803-0.971	0.846	0.816	0.889	0.912	0.774	0.586
Time Cohort 1	Model3	0.896	0.820-0.972	0.800	0.763	0.852	0.879	0.719	0.624
Time Cohort 2	C	0.772	0.672-0.872	0.756	0.796	0.703	0.780	0.722	0.756
Time Cohort 2	MD_Habitat	0.751	0.643-0.859	0.698	0.551	0.892	0.871	0.600	0.715
Time Cohort 2	MK_Habitat	0.836	0.754-0.918	0.756	0.633	0.919	0.912	0.654	0.739
Time Cohort 2	MK+MD_Habitat	0.890	0.821-0.959	0.860	0.837	0.892	0.911	0.805	0.526
Time Cohort 2	T	0.803	0.717-0.889	0.721	0.531	0.973	0.963	0.610	0.664
Time Cohort 2	T+C	0.839	0.757-0.921	0.779	0.694	0.892	0.895	0.688	0.671
Time Cohort 2	Model1	0.920	0.865-0.975	0.849	0.837	0.865	0.891	0.800	0.638
Time Cohort 2	Model2	0.891	0.822-0.960	0.837	0.857	0.811	0.857	0.811	0.539
Time Cohort 2	Model3	0.945	0.884-0.857	0.884	0.857	0.919	0.933	0.829	0.618
**Research on predicting LNM in Rectal cancer**									
**Cohort**	**Model**	**AUC**	**95%CI**	**Accuracy**	**Sensitivity**	**Specificity**	**PPV**	**NPV**	**Threshold**
Time Cohort 1	C_n	0.730	0.605-0.855	0.708	0.652	0.738	0.577	0.795	0.427

Time Cohort 1	MD_Habitat_n	0.694	0.567-0.821	0.738	0.261	1.000	1.000	0.712	0.489
Time Cohort 1	MK_Habitat_n	0.827	0.723-0.931	0.815	0.522	0.976	0.923	0.788	0.545
Time Cohort 1	MK+MD_Habitat_n	0.855	0.761-0.949	0.738	0.913	0.643	0.583	0.931	0.233
Time Cohort 1	T_n	0.768	0.656-0.880	0.708	0.957	0.571	0.550	0.960	0.405
Time Cohort 1	T+C_n	0.827	0.731-0.923	0.754	0.826	0.714	0.613	0.882	0.319
Time Cohort 1	Model1_n	0.903	0.811-0.995	0.862	0.913	0.833	0.750	0.946	0.297
Time Cohort 1	Model2_n	0.855	0.765-0.945	0.800	0.652	0.881	0.750	0.822	0.433
Time Cohort 1	Model3_n	0.916	0.847-0.985	0.877	0.913	0.857	0.778	0.947	0.325
Time Cohort 2	C_n	0.821	0.727-0.915	0.779	0.727	0.833	0.821	0.745	0.458
Time Cohort 2	MD_Habitat_n	0.744	0.644-0.844	0.721	0.773	0.667	0.708	0.737	0.721
Time Cohort 2	MK_Habitat_n	0.787	0.695-0.879	0.721	0.545	0.905	0.857	0.655	0.557
Time Cohort 2	MK+MD_Habitat_n	0.819	0.729-0.909	0.779	0.682	0.881	0.857	0.725	0.615
Time Cohort 2	T_n	0.748	0.646-0.850	0.686	0.568	0.810	0.758	0.642	0.470
Time Cohort 2	T+C_n	0.902	0.845-0.959	0.826	0.750	0.905	0.892	0.776	0.646
Time Cohort 2	Model1_n	0.906	0.849-0.963	0.837	0.682	1.000	1.000	0.750	0.758
Time Cohort 2	Model2_n	0.820	0.726-0.914	0.791	0.773	0.810	0.810	0.773	0.522
Time Cohort 2	Model3_n	0.910	0.853-0.967	0.826	0.659	1.000	1.000	0.737	0.790

***Note--**95%CI, 95% confidence interval; AUC, area under the curve; PPV, positive predictive value; NPV, negative predictive value; C, clinical model; T, MK+MD traditional radiomics model; T+C, MK+MD traditional radiomics model+clinical model; Model1, MK+MD_Habitat+C; Model2, MK+MD_Habitat+T; Model3, MK+MD_Habitat+C+T; Time Cohort 1, A patient cohort from September 2023 to May 2024;Time Cohort 2, A patient cohort from June 2024 to January 2025.
